# Genomic Copy Number Variations in the Genomes of Leukocytes Predict Prostate Cancer Clinical Outcomes

**DOI:** 10.1371/journal.pone.0135982

**Published:** 2015-08-21

**Authors:** Yan P. Yu, Silvia Liu, Zhiguang Huo, Amantha Martin, Joel B. Nelson, George C. Tseng, Jian-Hua Luo

**Affiliations:** 1 Department of Pathology, University of Pittsburgh School of Medicine, Pittsburgh, Pennsylvania, United States of America; 2 Department of Biostatistics, University of Pittsburgh School of Medicine, Pittsburgh, Pennsylvania, United States of America; 3 Department of Urology, University of Pittsburgh School of Medicine, Pittsburgh, Pennsylvania, United States of America; Innsbruck Medical University, AUSTRIA

## Abstract

Accurate prediction of prostate cancer clinical courses remains elusive. In this study, we performed whole genome copy number analysis on leukocytes of 273 prostate cancer patients using Affymetrix SNP6.0 chip. Copy number variations (CNV) were found across all chromosomes of the human genome. An average of 152 CNV fragments per genome was identified in the leukocytes from prostate cancer patients. The size distributions of CNV in the genome of leukocytes were highly correlative with prostate cancer aggressiveness. A prostate cancer outcome prediction model was developed based on large size ratio of CNV from the leukocyte genomes. This prediction model generated an average prediction rate of 75.2%, with sensitivity of 77.3% and specificity of 69.0% for prostate cancer recurrence. When combined with Nomogram and the status of fusion transcripts, the average prediction rate was improved to 82.5% with sensitivity of 84.8% and specificity of 78.2%. In addition, the leukocyte prediction model was 62.6% accurate in predicting short prostate specific antigen doubling time. When combined with Gleason’s grade, Nomogram and the status of fusion transcripts, the prediction model generated a correct prediction rate of 77.5% with 73.7% sensitivity and 80.1% specificity. To our knowledge, this is the first study showing that CNVs in leukocyte genomes are predictive of clinical outcomes of a human malignancy.

## Introduction

Prostate cancer is one of the leading causes of death for men in the United States. It has considerable heterogeneity in biological aggressiveness and clinical prognosis[1–3]. Since the implementation of serum PSA screening, the clinical detection rate of prostate cancer has been increased substantially due primarily to the identification of small, low grade cancers that would likely not progress[1]. Yet, close to 30,000 patients die of prostate cancer annually[4]. Accurate prediction of the aggressive behavior of prostate cancer remains elusive.

Currently, several treatment options are available for prostate cancer patients including watchful waiting, radiation, hormonal/chemo-therapy and radical prostatectomy. Gleason grading alone or in combination with other clinical indicators such as serum prostate specific antigen levels and pathological or clinical staging has been the guiding tool in selecting these treatment options. A significant number of prostate cancer patients, however, experienced recurrence after surgical resection of the prostate gland. There is clearly a need for better prediction of the prognosis of prostate cancer. Previous cytogenetic and other genome studies suggested a clear link between genome abnormalities and prostate cancer[5–21]. Recent analyses of genome copy number of prostate cancer, benign tissues adjacent to cancer and blood samples from prostate cancer patients suggested that genome deletion and amplification of certain regions in prostate cancer samples were associated with poor clinical outcomes[14; 22]. Whole genome and transcriptome sequencing revealed fusion transcripts in prostate cancer predictive of prostate cancer recurrence[23]. In this study, we performed whole genome copy number analyses on leukocytes from prostate cancer patients. Significant copy number variations (CNV) were identified in the genome of leukocytes of prostate cancer patients. We found that sizes of CNVs in leukocytes of prostate cancer samples were highly correlative to prostate cancer recurrence. Prediction models were built to predict prostate cancer outcomes based on the size of CNVs of the leukocytes.

## Materials and Methods

The protocol of the study was approved by University of Pittsburgh Institutional Review Board.

### Tissue processing, DNA extraction, amplicon generation, labeling, hybridization, washing and scanning of SNP 6.0 chips

Prostate cancer samples were obtained from University of Pittsburgh Medical Center Tissue Bank. These samples were collected from 1998–2012. Two hundred seventy-three buffy coat samples from prostate cancer patients were analyzed. Among these samples, 143 samples were followed at least 90 months, 35 patients were non-recurrent for 90 months or more, 55 patients experiencing recurrence with short PSADT (PSA doubling time <4 months), and 53 patients experiencing recurrence with long PSADT (PSA doubling time >15 months) after radical prostatectomy (S1 Table). The Gleason’s scores of all prostate cancer samples were reassessed by UPMC pathologists before the study. Clinical follow-up was conducted by office examination record, blood PSA survey and radiographic follow-up. These follow-ups were carried out for up to a 15 year period after the patient had a radical prostatectomy. The protocol was approved by “University of Pittsburgh Institutional Review Board”. Five hundred nanograms of genomic DNA were digested with Sty1 and Nsp1 for 2 hours at 37°C. The digested DNA was purified and ligated with primer/adaptors at 16°C for 12–16 hours. Amplicons were generated by performing PCR using primers provided by the manufacturer (Affymetrix, CA) on the ligation products using the following program: 94°C for 3 min, then 35 cycles of 94°C 30 second, 60°C for 45 sec and 65°C for 1 minute. This was followed by extension at 68°C for 7 min. The PCR products were then purified and digested with DNAseI for 35 min at 37°C to fragment the amplified DNA. The fragmented DNA was then labeled with biotinylated nucleotides through terminal deoxynucleotide transferase for 4 hours at 37°C. Two hundred fifty micrograms of fragmented DNA were hybridized with a pre-equilibrated Affymetrix chip SNP 6.0 at 50°C for 18 hours. Procedures of washing and scanning of SNP 6.0 chips followed the manuals provided by Affymetrix, Inc. Raw data information of SNP6.0 from these samples was deposited in “Gene Expression Omnibus” (GEO, accession number GSE70650).

### Statistical analysis

#### Copy number variation analysis

**Fig 1:**
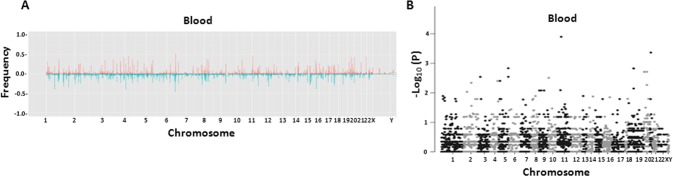
Copy number variations (CNV) in blood and prostate cancer from prostate cancer patients. (A) Histogram of frequency of amplification (red) or deletion (blue) of genome sequences of leukocytes (upper panel, n = 273) from prostate cancer patients. (B) Manhattan plots of p-values in association with prostate cancer recurrence of each gene CNV from leukocytes.

CEL files were analyzed with Genotyping Console for quality control analysis. Samples with QC call above 80% and QC contrast ratio above 0.4 were admitted into the analysis. To analyze CNV, CEL files were imported into Partek GenomeSuite 6.6 to generate copy number from raw intensity. To plot the histograms, deletion or amplification of genomes were analyzed by first limiting to the regions with p-value less than 0.001. The selected regions were subsequently filtered by limiting to the regions with at least 10 markers and 2 kb in size. The regions were then mapped to known genes. The frequencies of amplification and deletions were plotted to the genome corresponding to the gene locations (Fig 1A). For each gene, Fisher’s exact test was applied to test the association between CNV involvement and sample recurrence status. Then the minus log p-values were plotted on the Manhattan plot with their corresponding gene chromosome locations to generate Fig 1B. Benjamini-Hochberg (BH) method was applied to correct the p-values. The CNV-gene enriched pathways were selected by Kolmogorov-Smirnov test on the gene adjusted p-values. Pathway p-values were also corrected by BH method.

#### Machine learning methods to predict recurrent and fast-recurrent status

**Fig 2:**
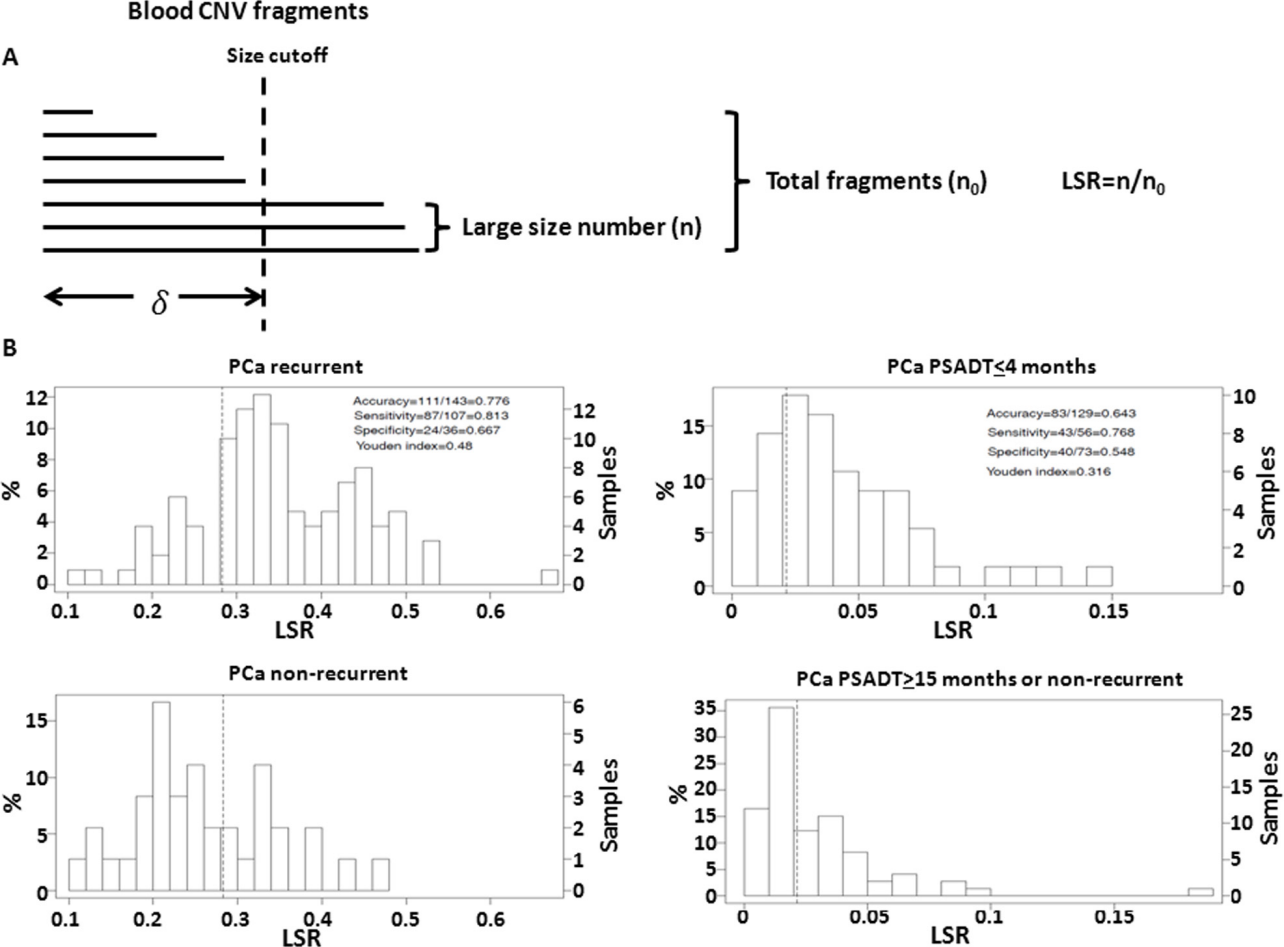
Large size ratio (LSR) of CNVs from leukocytes from prostate cancer patients are correlated with aggressive behavior of prostate cancer. (A) Schematic diagram of LSR model of leukocyte CNV. (B) LSRs from leukocytes are associated with aggressive prostate cancer recurrence behavior. Upper panel: Correlation of LSRs from leukocyte genomes with prostate cancers that were recurrent; Lower panel: Correlation of LSRs from leukocyte genomes with prostate cancers that were non-recurrent 90 months after radical prostatectomy. (C) LSRs from leukocytes are associated with short PSADT. Upper panel: Correlation of LSRs from leukocyte genomes with prostate cancers that had recurrent serum prostate specific antigen doubling time (PSADT) 4 months or less; Lower panel: Correlation of LSRs from leukocyte genomes with prostate cancers that were not recurrent or recurrent but having PSADT 15 months or more.

**Fig 3:**
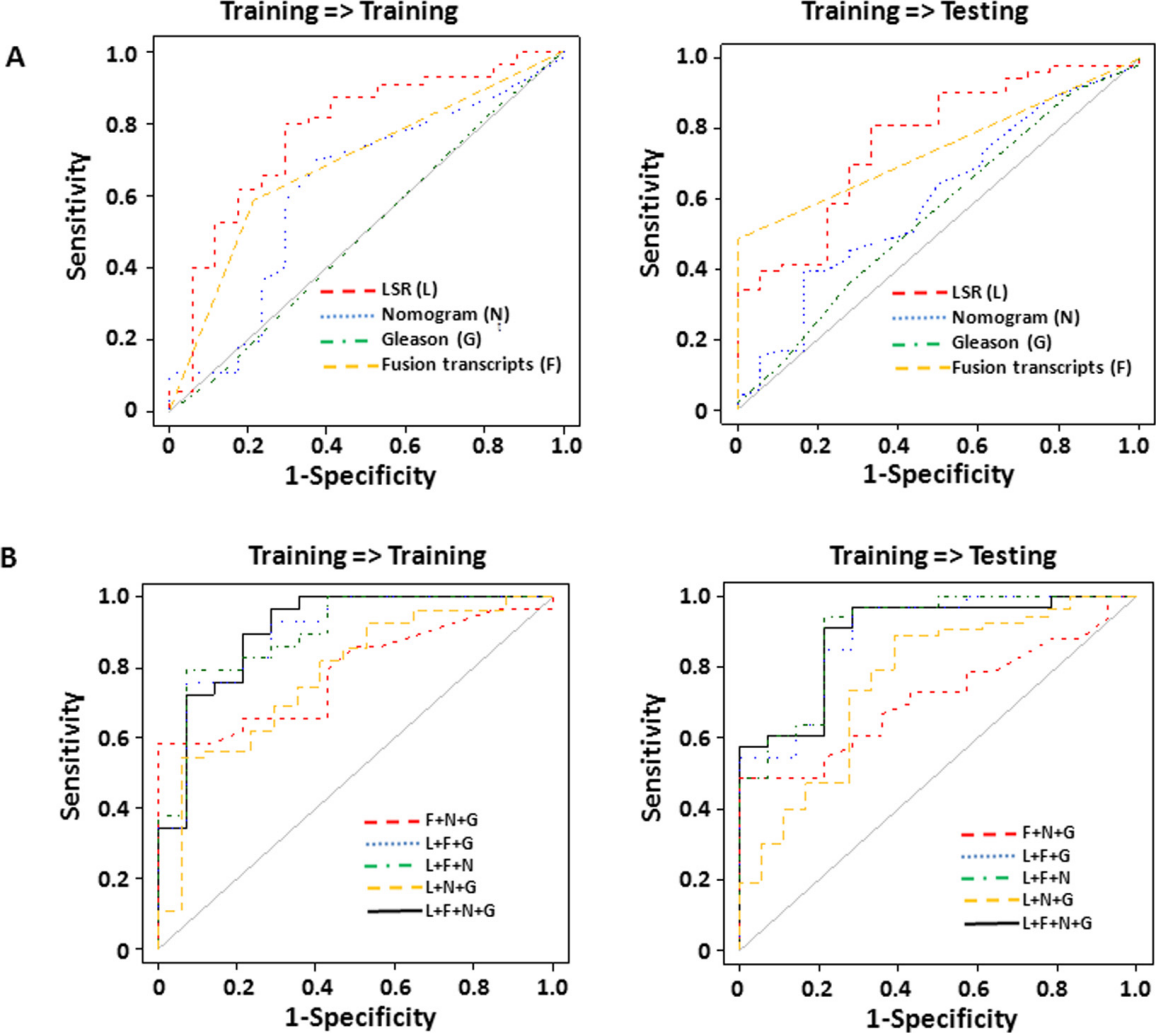
LSR of genome CNV from leukocytes to predict prostate cancer recurrence. (A) LSR derived from leukocyte genome CNV predicts prostate cancer recurrence. Receiver operating curve (ROC) analyses using LSRs derived from leukocyte CNVs as prediction parameter (red) to predict prostate cancer recurrence, versus Nomogram (blue), Gleason’s grade (green) and the status of 8 fusion transcripts[14] (yellow). The samples were equally split randomly into training and testing sets 10 times. The ROC analysis represents the results from the most representative split. (B) Combination of LSR (L), Gleason’s grade (G), Nomogram (N) and the status of fusion transcripts (F) to predict prostate cancer recurrence. ROC analysis of a model combining LSR, fusion transcripts, Nomogram and Gleason’s grade using LDA is indicated by black. ROC analysis of a model combining fusion transcripts, Nomogram and Gleason’s grade using LDA is indicated by red. ROC analysis of a model combining LSR, fusion transcripts and Gleason’s grade using LDA is indicated by blue. ROC analysis of a model combining LSR, fusion transcripts and Nomogram using LDA is indicated by green. ROC analysis of a model combining LSR, Nomogram and Gleason’s grade is indicated by yellow. Similar random splits of training and testing data sets were performed as of (A).

**Fig 4:**
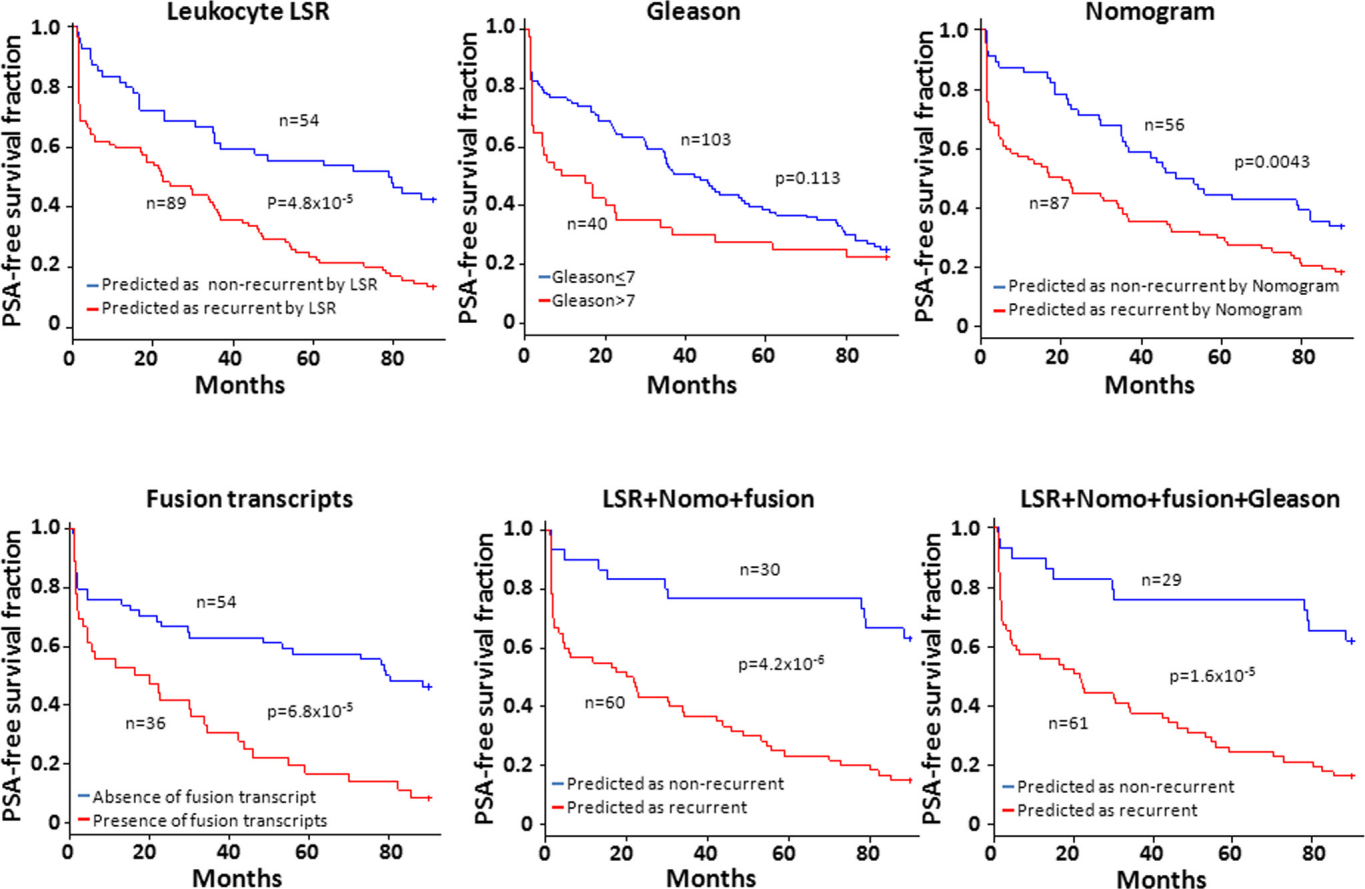
Large LSRs of genome CNVs from leukocytes correlated with lower PSA-free survival. Kaplan-Meier analysis on patients predicted by LSR based on CNV of patients’ leukocytes as likely recurrent versus likely non-recurrent (upper left). Similar survival analyses were also performed on case segregations based on Gleason’s grades (upper middle), Nomogram probability (upper right), the status of 8 fusion transcripts (lower left), or a model by combining LSR, Nomogram and fusion transcript status using LDA (lower middle), or a model by combining LSR, Nomogram, Gleason grade and fusion transcript status using LDA (lower right). Number of samples analyzed and p values are indicated.

**Fig 5:**
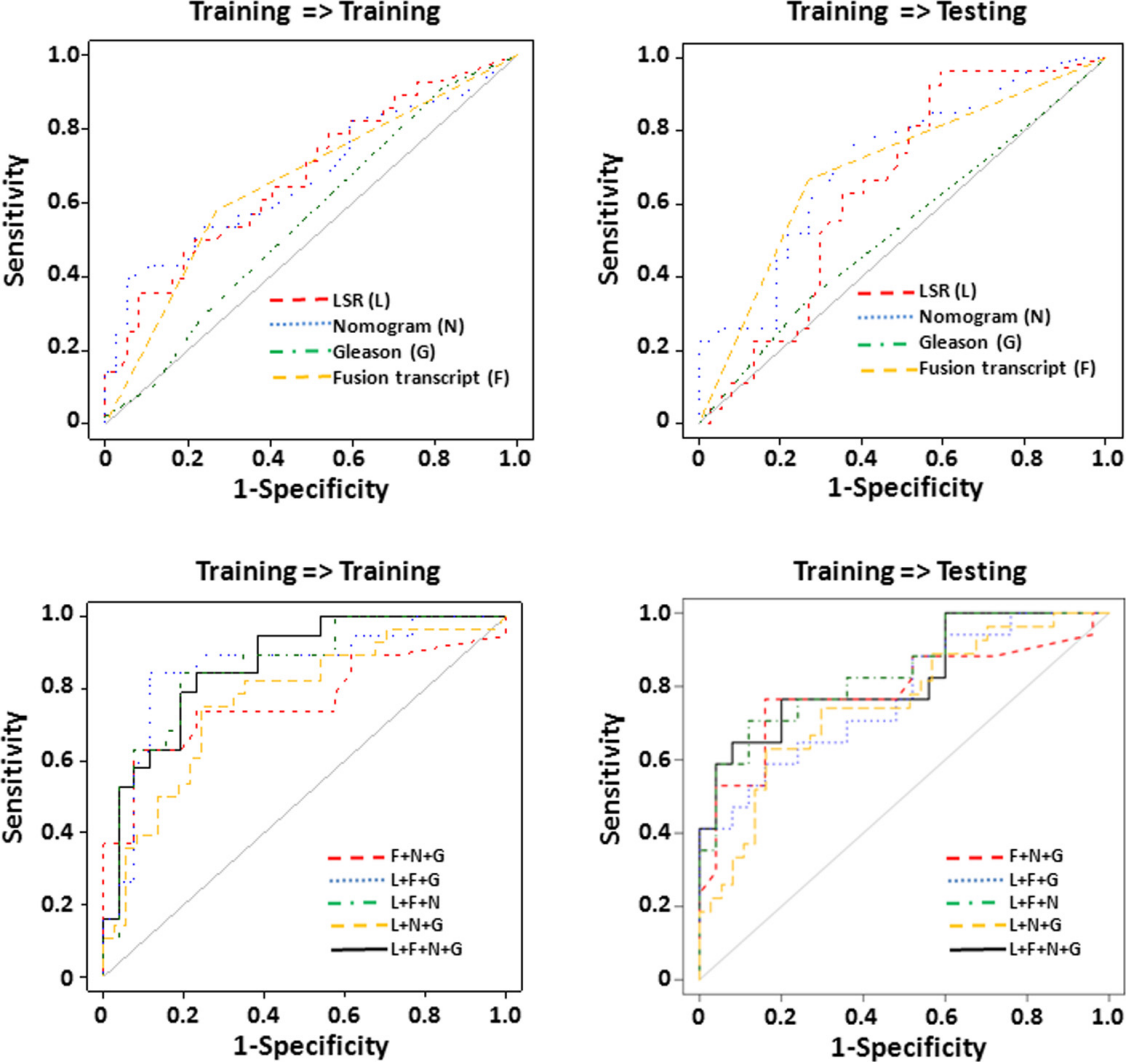
LSR of genome CNV from leukocytes to predict prostate cancer recurrence with short PSADT. LSR derived from leukocyte genome CNV predicts PSADT 4 months or less. ROC analysis using LSRs derived from leukocyte CNVs as a prediction parameter (red) to predict PSADT 4 months or less, versus Nomogram (blue), Gleason’s grade (green) and the status of 8 fusion transcripts[14] (yellow). Samples were analyzed by the same procedure as Fig 3. (B) Combination of LSR (L), Gleason’s grade (G), Nomogram (N) and the status of fusion transcripts (F) to predict prostate cancer recurrent PSADT 4 months or less. ROC analysis of a model combining LSR, fusion transcripts, Nomogram and Gleason’s grade using LDA is indicated by black. ROC analysis of a model combining fusion transcripts, Nomogram and Gleason’s grade using LDA is indicated by red. ROC analysis of a model combining LSR, fusion transcripts and Gleason’s grade using LDA is indicated by blue. ROC analysis of a model combining LSR, fusion transcripts and Nomogram using LDA is indicated by green. ROC analysis of a model combining LSR, Nomogram and Gleason’s grade is indicated by yellow.

**Fig 6:**
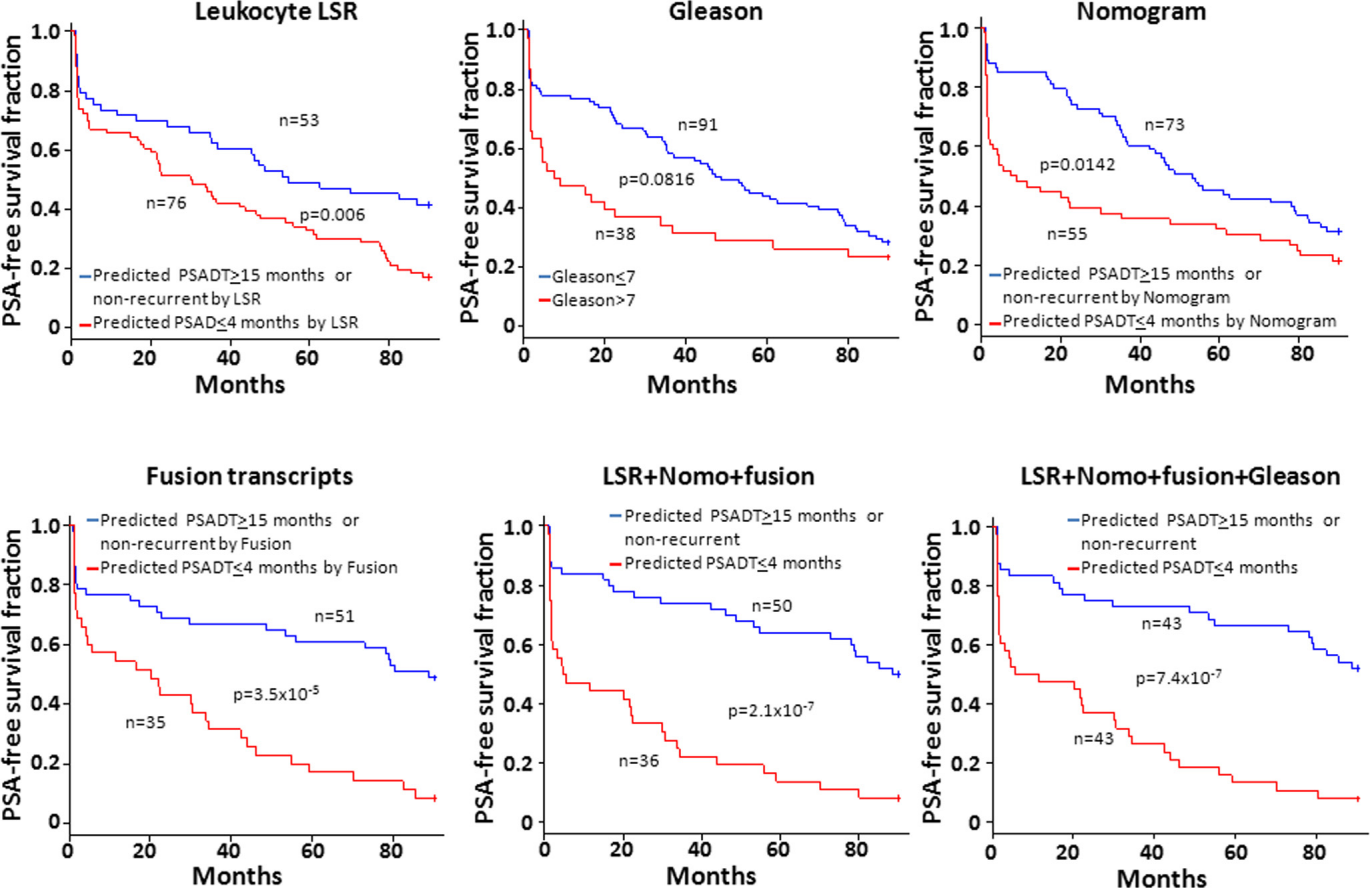
Genome CNVs from leukocytes predicting short PSADT correlated with lower PSA-free survival. Kaplan-Meier analysis on patients predicted by LSR based on CNV of patients’ leukocytes as likely recurrent and having PSADT 4 months or less versus likely non-recurrent or recurrent but having PSADT 15 months or more (upper left). Similar survival analyses were also performed on case segregations based on Gleason’s grades (upper middle), Nomogram probability (upper right), the status of 8 fusion transcripts (lower left), or a model by combining LSR, Nomogram and fusion transcript status using LDA (lower middle), or a model by combining LSR, Nomogram, Gleason grade and fusion transcript status using LDA (lower right). Number of samples analyzed and p values are indicated.

We constructed prediction models for two types of clinical comparisons: (1) non-recurrent versus recurrent; (2) non-fast recurrent (i.e. non-recurrent or recurrent but having prostate specific antigen doubling time [PSADT]≥15 months) versus fast-recurrent (recurrent PSADT≤ 4 months). For each comparison, the models were constructed using Gleason score (G), Nomogram score (N), fusion transcript status (F) or blood CNV information (L) separately. For Gleason score discrimination, we used binary prediction (0 meaning Gleason score ≤ 7 and 1 meaning Gleason score > 7). For Nomogram score, the 7 year survival probability obtained from http://www.mskcc.org/nomograms/prostate was used[24]. For fusion status, we applied eight fusion transcripts (TRMT11-GRIK2, SLC45A2-AMACR, MTOR-TP53BP1, LRRC59-FLJ60017, TMEM135-CCDC67, KDM4-AC011523.2, MAN2A1-FER and CCNH-C5orf30) previously identified and validated in a multi-center study[23]. A binary fusion score was used (0 meaning none of the eight fusions detected; 1 meaning one or more fusion transcripts detected). For prediction using gene CNV of leukocytes, we found little predictive power from gene-based association (Fig 1B). As a result, we developed a large size ratio (LSR) model based on the assumption that untargeted CNV aberrations in blood played a significant role in predisposing prostate tumors to aggressiveness. As shown in Fig 2A, LSR was defined as the proportion of large size CNV identified in the blood genome of a given patient, where large size was defined by threshold δ. In each two-fold cross-validation, samples were randomly and equally split into two data sets. In the first dataset treated as training data, the best δ parameter in LSR model and the best cutoffs of Nomogram and LSR scores were selected by maximizing the highest AUC (area under the curve) and Youden index (i.e. sensitivity+specificity-1). The models were then applied to the second dataset as testing data. The cross-validation was then repeated using the second dataset as training data and the first dataset as test data. ROC curves were plotted by varying the cutoffs in both the training and testing datasets. The corresponding overall accuracy, sensitivity, specificity, Youden index and AUC were calculated to evaluate the performance. The equal-splitting validation was repeated for 14 times and the top 2 and bottom 2 splitting with the highest and lowest sum of AUCs were removed to avoid accidentally extreme training/testing assignment. The remaining 10 cross-validation results were finally averaged (Table 1 and Table 2). ROC and Kaplan-Meier survival curves in Figs 3–6 are the representative results of the 10 predictions closest to the averaged values.

**Table 1:** Prediction of prostate cancer recurrence based on leukocyte LSR, Gleason, Nomogram and fusion transcript status.

Model	Accuracy	Sensitivity	Specificity	Youden index	AUC	ROC p-value
**Equal split training data (n = 72)**						
LSR	0.765	0.778	0.724	0.502	0.779	2.15 x 10^−5^
Nomogram	0.66	0.675	0.612	0.286	0.63	3.67 x 10^−2^
Gleason	0.403	0.296	0.747	0.043	0.538	3.28 x 10^−1^
Fusion	0.642	0.537	0.897	0.434	0.717	5.84 x 10^−4^
L+N+F	0.864	0.856	0.885	0.742	0.917	2.12 x 10^−13^
L+N+G	0.768	0.767	0.771	0.538	0.803	1.69 x 10^−6^
N+F+G	0.751	0.698	0.87	0.568	0.799	3.05 x 10^−5^
L+F+G	0.863	0.867	0.85	0.717	0.91	3.33 x 10^−12^
L+N+F+G	0.879	0.888	0.854	0.742	0.923	3.75 x 10^−14^
**Equal split testing data (n = 71)**						
LSR	0.739	0.768	0.656	0.423	0.76	1.38 x 10^−4^
Nomogram	0.613	0.653	0.494	0.147	0.589	1.93 x 10^−1^
Gleason	0.394	0.277	0.739	0.016	0.513	3.52 x 10^−1^
Fusion	0.647	0.53	0.892	0.422	0.711	9.11 x 10^−4^
L+N+F	0.786	0.839	0.678	0.517	0.879	4.19 x 10^−9^
L+N+G	0.692	0.719	0.611	0.33	0.722	1.77 x 10^−3^
N+F+G	0.64	0.641	0.65	0.292	0.709	8.82 x 10^−3^
L+F+G	0.76	0.812	0.66	0.472	0.856	1.61 x 10^−7^
L+N+F+G	0.757	0.817	0.64	0.457	0.853	3.94 x 10^−7^

L-LSR; N-Nomogram; F-fusion transcript status; G-Gleason grade.

L+N+F: LDA model to combine LSR, Nomogram and fusion transcript status

L+N+G: LDA model to combine LSR, Nomogram and Gleason grade

N+F+G: LDA model to combine Nomogram, fusion transcript status and Gleason grade

L+N+F+G: LDA model to combine LSR, Nomogram, fusion transcript status and Gleason grade.

The results represent the average of the analyses on 10 random equal splits of training and testing results.

**Table 2:** Prediction of prostate cancer recurrent PSADT≤4 months based on leukocyte LSR, Gleason, Nomogram and fusion transcript status.

Model	Accuracy	Sensitivity	Specificity	Youden index	AUC	ROC p-value
**Equal split training data (n = 65)**						
LSR	0.655	0.739	0.592	0.331	0.662	1.63 x 10^−2^
Nomogram	0.678	0.593	0.743	0.336	0.676	8.19 x 10^−3^
Gleason	0.423	0.3	0.743	0.043	0.55	4.63 x 10^−1^
Fusion	0.688	0.626	0.725	0.351	0.676	1.89 x 10^−2^
L+N+F	0.825	0.788	0.85	0.638	0.86	8.00 x 10^−9^
L+N+G	0.728	0.779	0.689	0.468	0.743	1.97 x 10^−4^
N+F+G	0.791	0.71	0.845	0.555	0.794	2.55 x 10^−4^
L+F+G	0.809	0.822	0.798	0.62	0.839	5.34 x 10^−7^
L+N+F+G	0.83	0.806	0.846	0.652	0.866	5.29 x 10^−9^
**Equal split testing data (n = 64)**						
LSR	0.595	0.636	0.564	0.2	0.66	1.67 x 10^−2^
Nomogram	0.645	0.611	0.67	0.281	0.707	1.39 x 10^−3^
Gleason	0.445	0.324	0.754	0.078	0.532	5.68 x 10^−1^
Fusion	0.684	0.613	0.731	0.344	0.672	1.96 x 10^−2^
L+N+F	0.736	0.669	0.782	0.451	0.799	4.84 x 10^−5^
L+N+G	0.65	0.678	0.63	0.308	0.715	1.45 x 10^−3^
N+F+G	0.699	0.598	0.764	0.362	0.764	5.97 x 10^−4^
L+F+G	0.698	0.668	0.723	0.39	0.768	4.79 x 10^−4^
L+N+F+G	0.72	0.667	0.756	0.423	0.788	1.26 x 10^−4^

L-LSR; N-Nomogram; F-fusion transcript status; G-Gleason grade.

L+N+F: LDA model to combine LSR, Nomogram and fusion transcript status

L+N+G: LDA model to combine LSR, Nomogram and Gleason grade

N+F+G: LDA model to combine Nomogram, fusion transcript status and Gleason grade

L+N+F+G: LDA model to combine LSR, Nomogram, fusion transcript status and Gleason grade.

The results represent the average of the analyses on 10 random equal splits of training and testing results.

To test whether combining multiple data information improves the prediction result, we applied linear discriminant analysis (LDA) to combine two or more predictive factors. All possible combinations were performed. Models using (1) L+N+F (2) L+N+G (3) N+F+G (4) L+F+G (5) L+N+F+G are shown in Figs 3 and 5.

#### Kaplan-Meier curve analysis

For the survival evaluation (Figs 4 and 6), we combined the two-fold cross validation of “Training = >Testing” result to compare the performance of different methods, except for Gleason score that we used (≤7 VS >7 as cut-off for the whole samples). Kaplan-Meier curves were truncated at 90 months follow-up. Log-rank test was performed to calculate the p-value between survival curves of two predicted outcomes. To evaluate whether the survival difference for one model was significantly better than the other, we define a test statistics U as the absolute difference of the log-rank test statistics from the two models. Theoretically under the null hypothesis (two models were non-discriminant), the test statistics U followed a distribution of absolute difference of two independent chi-squared (degree of freedom = 1) distributions. As a result, we sampled 10,000,000 times from the absolute difference of two independent chi-squared distributions to form null distribution and evaluate the p-values.

## Results

Genome copy abnormalities are some of the hallmarks for prostate cancer. However, little is known about the genome copy abnormalities in non-cancerous tissues from prostate cancer patients. To analyze the regions of amplification and deletion in the genome of leukocytes from prostate cancer patients, 273 buffy coats from prostate cancer patients were analyzed for CNV across the entire genome using Affymetrix SNP6.0. Using the cutoff criteria of size ≥2 Kb, marker number ≥ 10 and p<0.001, a total of 41589 CNV fragments were identified, including 24213 segments of deletion and 17376 of amplification, involving 17865 genes based on the Partek gene annotation (Fig 1A). This translates to an average of about 152 CNVs per sample. The average size of CNV in the genome of the leukocytes is about 147 Kb. On average, 256 genes were found to have either copy number gain or loss per genome. Among the 273 blood samples, 143 blood samples have more than 90 months of clinical follow-ups in terms of prostate cancer recurrence. Interestingly, when categorizing the blood samples based on the status of prostate cancer recurrence, CNV of leukocytes from patients who experienced recurrence after radical prostatectomy had an average of >3.2 fold larger size of CNV versus CNV from patients who had no recurrence for at least 90 months. Two-sided t test showed a strong correlation between the size of CNV in leukocytes and prostate cancer recurrence (p = 2.2 x 10^−16^), suggesting that the size of germ line CNV may play a significant role in predisposing prostate cancer to aggressive clinical courses. However, no specific (FDR = 0.05) gene involved in CNV of genome fragment reaches the threshold that differentiates recurrent prostate cancer versus those of non-recurrent (Fig 1B). Together, the results indicate that the gene-based prediction model is unlikely to succeed in the leukocyte CNV analysis but size distribution of CNVs can be predictive.

To examine whether germ line CNV is predictive of recurrence of prostate cancer, an algorithm utilizing ratios of the number of large size fragments was developed. As illustrated in Fig 2A, for each sample, large size ratio (LSR) is defined as the ratio of CNV fragments whose sizes are greater than a size cutoff (δ) over the total number of CNV fragments. For example, 3 of the 7 detected CNVs in Fig 2A are found “large size fragments” (size ≥ δ) and the LSR of this patient is calculated as 3/7 = 0.43. In Fig 2B, the distribution of LSR from patients who experienced prostate cancer recurrence showed significantly higher values than those who did not experience recurrence. Similarly, the distribution of LSR from patients with fast recurrence (PSADT≤ 4 months) was significantly higher than those from non-fast recurrent patients (non-recurrent or recurrent but having PSADT≥ 15 months, Fig 2C). In the LSR model, the size threshold δ is determined by maximizing the AUC. When δ values were optimized (S1 Fig, δ = 10^4.5^ = 31622 bp for recurrent prediction model and 1B selected δ = 10^5.7^ = 501187 bp for fast recurrent prediction), it predicts prostate cancer recurrence with accuracy of 77.6%, with sensitivity of 80.4% and specificity of 68.6%, while fast recurrence with accuracy of 62.4%, with sensitivity of 72.9% and specificity of 54.1%.

To validate this model, 143 blood samples (S1 Table) from prostate cancer patients were randomly split into a training set (72 samples) and a testing set (71 samples). The optimized large-size cutoff δ and LSR-cutoff were obtained from the training analysis by maximizing the Youden index. The parameters were then applied to the testing data set to assess the prediction accuracy. The validation was then repeated 14 times and the best 2 and worst 2 were removed to avoid extreme randomization. The remaining 10 results from these training and testing analyses were averaged (Table 1). As shown in Fig 3A (representative analyses in S2 Table) and Table 1, the training accuracy of LSR model in predicting prostate cancer recurrence reaches 76.5%, with 77.8% sensitivity and 72.4% specificity. When the parameters were applied to the testing set, the prediction accuracy reaches 73.9%, with 76.8% sensitivity and 65.6% specificity. These prediction rates are better than those of Nomogram (66.0% accuracy for training and 61.3% for testing, Table 1), and are significantly higher than those of Gleason grade’s with single cutoff (40.3% for training and 39.4% for testing; p = 8.6x10^-3^ for training and p = 5.8x10^-3^ for testing by ROC comparison, see Table 1 and S3 Table).

To examine whether combination of different modalities will improve the prediction model, blood LSR, Nomogram, Gleason’s grade and the status of 8 fusion transcripts (TRMT11-GRIK2, SLC45A2-AMACR, MTOR-TP53BP1, LRRC59-FLJ60017, TMEM135 –CCDC67, KDM4-AC011523.2, MAN2A1-FER and CCNH-C5orf30)[23] in the prostate cancer samples were combined through linear discriminant analysis (LDA) to train the prediction model in the training set. Such model generated a prediction accuracy of 87.9%, with 88.8% sensitivity and 85.4% specificity for prostate cancer recurrence in the training set, and accuracy of 75.7%, with 81.7% sensitivity and 64.0% specificity in the testing set (Fig 3B and Table 1). Interestingly, the combination of LSR, Nomogram and the status of fusion transcripts appears to produce the best prediction results: 86.4% accuracy in the training set and 78.6% accuracy in the testing set. These prediction rates appear significantly better than those generated from any single modality (Table 1). To evaluate the contribution of each of these modalities to the combination model, subtraction of one of each modality at a time was made on the model to evaluate their impacts respectively. As shown in Fig 3B and Table 1, subtraction of LSR modality appeared to have the most significant impact on prediction of prostate cancer recurrence: The prediction accuracy rates drop from 87.9% to 75.1% (ROC p = 0.044, see S3 Table) in the training sets and from 75.7% to 64.0% (ROC p = 0.037) in the testing sets. This was followed by fusion genes (p-value between the two ROC curves was 0.109 for training and 0.159 for testing). On the other hand, subtraction of Nomogram or Gleason grade had no appreciable impact on the prediction performance of the model (Table 1, Fig 3 and S3 Table).

To examine the prediction performance of LSR score on PSA-free survival of prostate cancer patients, Kaplan-Meier analyses were performed on 143 patients who had definitive clinical information (S1 Table). Recurrence status for testing samples were predicted by the model trained from the training set, and the prediction model of training samples was trained from testing set. The merged two-fold cross-validation prediction results were used to divide the 143 patients into predicted recurrent group and non-recurrent group. As shown in Fig 4, when patients were predicted by LSR as high risk for prostate cancer recurrence, only 12.1% of the patients survived for 90 months without recurrence, while over 52.3% patients with LSR model predicted to be likely non-recurrent survived 90 months without any sign of recurrent prostate cancer (average p = 9.9 x 10^−5^ by log-rank test, Fig 4 and S4 Table). In contrast, Gleason score failed to produce statistically significant different results for recurrent and non-recurrent groups (p = 0.113 by log-rank test). Nomogram, however, generated statistically significant better clinical outcomes (33.9% versus 18.4% survival rate and p = 0.0038 for log-rank test) when patients were segregated based on predicted recurrent versus non-recurrent by Nomogram. When fusion transcripts, leukocyte genome LSR and Nomogram were combined, it improved the outcomes of prostate cancer prediction to 58.1% PSA-free survival if they were predicted to be non-recurrent by the model versus 16.9% if they were predicted as likely recurrent by the combined model (p = 2.9x10^-6^ for the two survival curves). This combined-modality model significantly outperforms any single modality prediction model (p = 6.6x10^-3^ versus LSR, p = 1.8x10^-5^ versus Gleason, p = 3.5x10^-4^ versus Nomogram, p = 0.017 versus fusion transcripts, see S5 Table). When Gleason grading was added to model, it did not improve the accuracy of prediction, but improved the survival curves.

Prostate cancer related death is closely associated with rising velocity of recurrent seral PSA. Short PSADT (<4 months) had been used as a surrogate for prostate cancer related death for the last 15 years[25; 26]. To examine whether LSR in the genome of leukocytes is also predictive of short PSADT, blood samples (S1 Table) were randomly split into training (65 samples) and testing (64 samples) sets. Similar processes were performed on these samples as described in recurrence prediction. As shown in Table 2, the LSR model in the training and testing data sets yielded an accuracy of prediction of PSADT = <4 months as 67.7% and 57.5%, respectively. The ROC curve of LSR model versus the diagonal line (random guess) has p-value = 0.016 for the training set and 0.017 for the testing set (Fig 5, Table 2 and S6 Table). The prediction based on Gleason scores yielded 42.3% accuracy for training set, and 44.5% for the testing data set. On the other hand, Nomogram generated a prediction accuracy of 67.8% and ROC p-value of 0.0082 in the training set and 64.5% accuracy and 0.0014 ROC p-value in the testing set. The status of fusion transcripts in the prostate cancer samples produced an accuracy of 68.8% and 68.4% in training and testing data sets, respectively. These 4 methods did not appear to be significantly better than one another when pairwise proportion tests were performed. However, when all 4 methods were combined, it yielded an accuracy of 83.0% (ROC p = 5.3 x 10^−9^) for the training set and 72.0% (ROC p = 1.3x10^-4^) for the testing set. These results were better than any single prediction modality in terms of accuracy, AUC and Youden Index values (Table 2). To investigate the impact of each of these modalities on the prediction model, each modality was individually subtracted from the combined prediction model. The prediction results showed a range of 72.8–82.5% accuracy in the training data set and 65.0–73.6% accuracy in the testing data set, when one modality was subtracted. Interestingly, when either blood LSR or cancer fusion transcript status was subtracted, the combined models yielded no significantly better predictions than any single modality prediction except Gleason’s (S7 Table), suggesting that blood LSR and fusion transcript status were the most significant contributors in the combined prediction model.

To analyze the impact of short PSADT prediction on prostate cancer PSA-free survivals, Kaplan-Meier analyses were performed on samples segregated based on the PSADT prediction by leukocyte genome LSR. As shown in Fig 6 and S8 Table, when samples predicted by blood LSR to have PSADT≤4 months, the PSA-free survival rate was 17.1% at 90th-month after radical prostatectomy, while the survival rate improved to 41.5% for those predicted to have PSADT≥15 months or non-recurrent (log-rank test p = 0.0039, see Fig 6 and S8 Table). In contrast, survival curves predicted by Gleason score ended up with similar survival rate at 90-month, and the p-value between two curves was 0.0816 by log-rank test. Nomogram had the PSA-free survival rate of 21.4% when patients were predicted to have PSADT≤4 months. This survival rate was 31.5% when patients were predicted to be non-recurrent (p = 0.0021 by log-rank test). However, when the model combining Gleason, Nomogram, fusion transcripts and blood LSR was applied, the PSA-free survival rate was only 7.9% when patients were predicted to have PSADT≤4 months, while the survival rate was 52.1% when the patients were predicted to have PSADT>4 months or non-recurrent (p = 1.6x10^-7^). The model combining 4 modalities significantly outperformed the prediction models based on Gleason grade (p = 1.5x10^-6^) or Nomogram (p = 3.0x10^-5^) or LSR (p = 1.9x10^-5^) or fusion transcripts (p = 0.0018) alone (S9 Table). These analyses clearly indicate that the sizes of copy number variation of human leukocytes are correlative with clinical behavior of prostate cancer. The combination of the genome CNV of leukocytes with clinical information of prostate cancer patients would yield much improved prediction models for prostate cancer behavior.

## Discussion

Extensive presence of CNV is one of the important features of human malignancies. CNV in normal tissues of healthy individuals was also well documented[14; 27; 28]. Since CNV analysis is largely insensitive to small contamination, it may require more than 25% contamination to detect an alteration of copy number in the genome. Small contamination of the blood stream by prostate cancer cells is generally undetected. The CNVs detected from the buffy coats in our study probably represent the genome CNVs from leukocytes. Our studies suggest that the sizes of CNV from leukocytes of prostate cancer patients are highly correlative with the clinical outcomes of prostate cancer. These CNVs spreads across all the chromosomes. Most of these CNVs overlap with the gene coding sequences of the genome. Interestingly, neither specific CNV fragment nor gene involved by these CNVs is significantly associated with the outcome of prostate cancer, suggesting that the impact of CNVs on prostate cancer is of collective nature. However, pathway analysis on genes that were involved in leukocyte genome CNV revealed enrichment of olfactory signaling pathways in recurrent-high risk patients from REACTOME (adjusted p = 5.0x10^-10^ using Kolmogorov-Smirnov test) and KEGG (adjusted p = 6.9x10^-10^) databases. The significance of leukocyte genome CNV enriched in this pathway is not clear. A recent study also suggests that higher copy number of mitochondria DNA is associated with the risk of prostate cancer. But it is unclear whether mitochondria DNA copy number is correlated with prostate cancer metastasis[29]. There is no clear link of leukocyte CNV with the severity of infiltrating lymphocytes in the prostate cancer samples.

The widespread and sporadic nature of these CNVs indicates that the leukocyte CNVs are of germline origin. As a result, our study implies that high numbers of large size germline CNVs predispose prostate cancer to aggressive behavior. These large size CNVs frequently overlap with multiple genes. The larger the size of the CNV is, the higher the number of genes could be impacted, and thus more metabolic and signaling pathways would be hit. Interestingly, one of the most frequent genes detected in large size CNVs is UDP glucuronosyltransferase 2 family, polypeptide B17 (UGT2B17). This gene encodes an enzyme responsible for transferring of glucuronic acid from uridine diphosphoglucuronic acid to a diverse array of substrates including steroid hormones and lipid-soluble drugs. UGT2B17 is essential for steroid metabolism. Genome deletion of UGT2B17 is associated with higher testosterone level[30]. As a result, germline CNV of UGT2B17 may have an impact on sex hormone metabolism, and thus affects the clinical course of prostate cancer. The expression levels of genes involved in CNV may be altered even in normal cells due to higher or lower copy number of the transcription units. Such subtle alterations could be exacerbated when cells become malignant because of the loss of the off-set mechanism. Indeed, higher numbers and larger sizes of CNVs and bigger CNV burden in prostate cancer samples are correlative with prostate cancer aggressiveness[14; 31]. As a result, germline CNV is possibly a pre-condition and down-stream mechanism leading to aggressive behavior of prostate cancer.

Prostate cancer is highly heterogeneous with various clinical outcomes. Most prostate cancers do not develop into life-threatening disease. Only a small fraction of prostate cancers are lethal and require aggressive treatment. When prostate cancer samples were segregated as likely lethal (recurrence occurred ≤12 months after radical prostatectomy and PSDAT≤4 months) versus those with no recurrence at all for 90 months, leukocyte LSR correctly predicted 78.3% accuracy with 73.9% sensitivity and 82.9% specificity for training and 66.9% accuracy with 59.4% sensitivity and 73.9% specificity for testing (S10–S14 Tables, S2 and S3 Figs). The model combining leukocyte LSR with Nomogram and fusion transcript status has an accuracy of 95.7% with 96.6% sensitivity and 94.7% specificity for training and an accuracy of 82.9% with 79.6% sensitivity and 85.5% specificity for testing. The multi-modality model outperformed all model based on single criteria in judging the lethality of prostate cancer.

Gleason’s grading has been the mainstay in judging the potential behavior of prostate cancer for many years. The accuracy of Gleason’s prediction is generally good when Gleason’s grade is high (8 and above). However, the prediction rates for prostate cancers with mid-range scores such as 7, are much less accurate. Furthermore, final Gleason’s grades cannot be determined until the entire prostate gland is examined. Thus, the determination of treatment modality of prostate cancer could be problematic. Even though genomic or epigenomic analyses of cancer cells from the blood[32] or from prostate[14; 33; 34] can offer significant insight into the prognosis of prostate cancer, leukocyte CNV represents the most non-invasive and least laborious approach to assess the metastatic potential of cancer. Conceivably, leukocyte CNV analysis offers an attractive alternative model in predicting prostate cancer clinical outcomes. There are several salient potentials for clinical application using the leukocyte CNV tests: For a patient being diagnosed of prostate cancer, CNV analysis done on the blood samples from the patient would eliminate the need for additional invasive procedure to decide a treatment mode. For a patient already having a radical prostatectomy, the CNV analysis on the blood sample, combined with information of fusion transcript status and Nomogram, may help to decide whether additional treatment is warranted to prevent prostate cancer recurrence. Since the leukocyte genome CNV test required no prostate cancer sample, it would be extremely useful if a patient has only a limited number of prostate cancer cells and Gleason’s grading or other pathological features cannot be determined. The only limitation of leukocyte CNV test is its slightly higher cost. In addition, the leukocyte CNV test is highly complement to clinical prediction parameters such as Gleason’s grade and Nomogram, and it enhances the prediction precision of these clinical parameters. As a result, the CNV analysis on the genome of leukocytes of prostate cancer patients may hold promise to become an important way to predict the behavior of prostate cancer.

## Supporting Information

S1 Fig Correlation of area under the curve (AUC) with LSR in predicting prostate cancer recurrence (left panel) or in predicting recurrent PSADT≤4 months (right panel). (TIF)

S2 Fig LSR of genome CNV from leukocytes to predict prostate cancer likely lethality. (A) LSR derived from leukocyte genome CNV predicts prostate cancer likely lethality (recurrent within 12 months of radical prostatectomy and PSADT≤4 months). Receiver operating curve (ROC) analyses using LSRs derived from leukocyte CNVs as prediction parameter (red) to predict prostate cancer likely lethality, versus Nomogram (blue), Gleason’s grade (green) and the status of 8 fusion transcripts[14] (yellow). The samples were equally split randomly into training and testing sets 10 times. The ROC analysis represents the results from the most representative split. (B) Combination of LSR (L), Gleason’s grade (G), Nomogram (N) and the status of fusion transcripts (F) to predict prostate cancer likely lethality. ROC analysis of a model combining LSR, fusion transcripts, Nomogram and Gleason’s grade using LDA is indicated by black. ROC analysis of a model combining fusion transcripts, Nomogram and Gleason’s grade using LDA is indicated by red. ROC analysis of a model combining LSR, fusion transcripts and Gleason’s grade using LDA is indicated by blue. ROC analysis of a model combining LSR, fusion transcripts and Nomogram using LDA is indicated by green. ROC analysis of a model combining LSR, Nomogram and Gleason’s grade is indicated by yellow. Similar random splits of training and testing data sets were performed as of (A).
(TIF)

S3 Fig Large LSRs of genome CNVs from leukocytes correlated with lower PSA-free survival. Kaplan-Meier analysis on patients predicted by LSR based on CNV of patients’ leukocytes as likely lethal (recurrent within 12 months of radical prostatectomy and PSADT≤4 months) versus likely non-recurrent (upper left). Similar survival analyses were also performed on case segregations based on Gleason’s grades (upper middle), Nomogram probability (upper right), the status of 8 fusion transcripts (lower left), or a model by combining LSR, Nomogram and fusion transcript status using LDA (lower middle), or a model by combining LSR, Nomogram, Gleason grade and fusion transcript status using LDA (lower right). Number of samples analyzed and p values are indicated.
(TIF)

S1 Table Clinical information for 143 blood samples. (DOCX)

S2 Table Prediction of prostate cancer recurrence based on leukocyte LSR, Gleason, Nomogram and fusion transcript status (the representative result for Fig 3). (DOCX)

S3 Table Pairwise ROC p-value for prostate cancer recurrent status prediction (the geometric mean of the 10 cross-validations). (DOCX)

S4 Table Survival p-values for the predicted prostate cancer recurrent and non-recurrent groups (the geometric mean of the 10 cross-validations). (DOCX)

S5 Table Pairwise survival p-value for prostate cancer recurrent status prediction (the geometric mean of the 10 cross-validations). (DOCX)

S6 Table Prediction of prostate cancer recurrent PSADT≤4 months based on leukocyte LSR, Gleason, Nomogram and fusion transcript status (the representative result for Fig 5). (DOCX)

S7 Table Pairwise ROC p-value for prostate cancer fast-recurrent status prediction (the geometric mean of the 10 cross-validations). (DOCX)

S8 Table Survival p-values for the predicted prostate cancer fast-recurrent and non-fast-recurrent groups (the geometric mean of the 10 cross-validations). (DOCX)

S9 Table Pairwise survival p-value for prostate cancer fast-recurrent status prediction (the geometric mean of the 10 cross-validations). (DOCX)

S10 Table Prediction of lethal prostate cancer recurrent (PSADT≤4 months and relapse time ≤12 months) VS non-recurrence based on leukocyte LSR, Gleason, Nomogram and fusion transcript status (the average result). (DOCX)

S11 Table Prediction of lethal prostate cancer recurrent (PSADT≤4 months and relapse time ≤12 months) VS non-recurrence based on leukocyte LSR, Gleason, Nomogram and fusion transcript status (the representative result for S2 Fig). (DOCX)

S12 Table Pairwise ROC p-value for prostate cancer lethal-recurrent and non-recurrent status prediction (the geometric mean of the 10 cross-validations). (DOCX)

S13 Table Survival p-values for the predicted prostate cancer lethal-recurrent and non-recurrent groups (the geometric mean of the 10 cross-validations). (DOCX)

S14 Table Pairwise survival p-value for prostate cancer lethal-recurrent and non-recurrent status prediction (the geometric mean of the 10 cross-validations). (DOCX)
