# Supplementary figures and images for: Genomic Copy Number Variations in the Genomes of Leukocytes Predict Prostate Cancer Clinical Outcomes
Source: PLoS One. 2015 Aug 21;10(8):e0135982. doi: 10.1371/journal.pone.0135982 (PMC4546524; doi:10.1371/journal.pone.0135982)

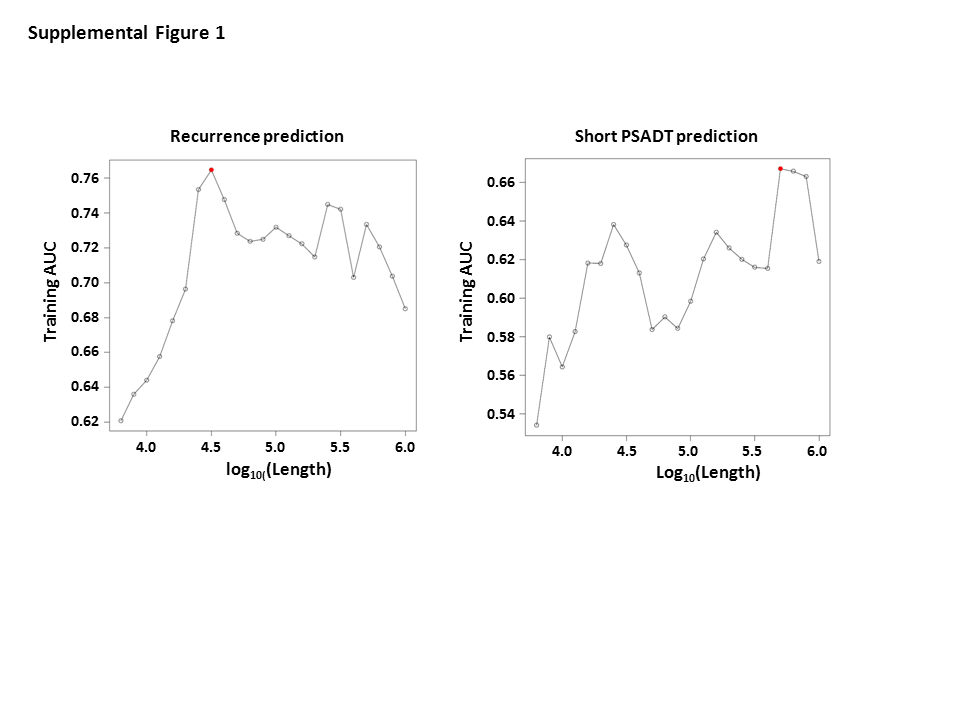

Supplement: S1 Fig — (TIF) [file pone.0135982.s001.TIF]

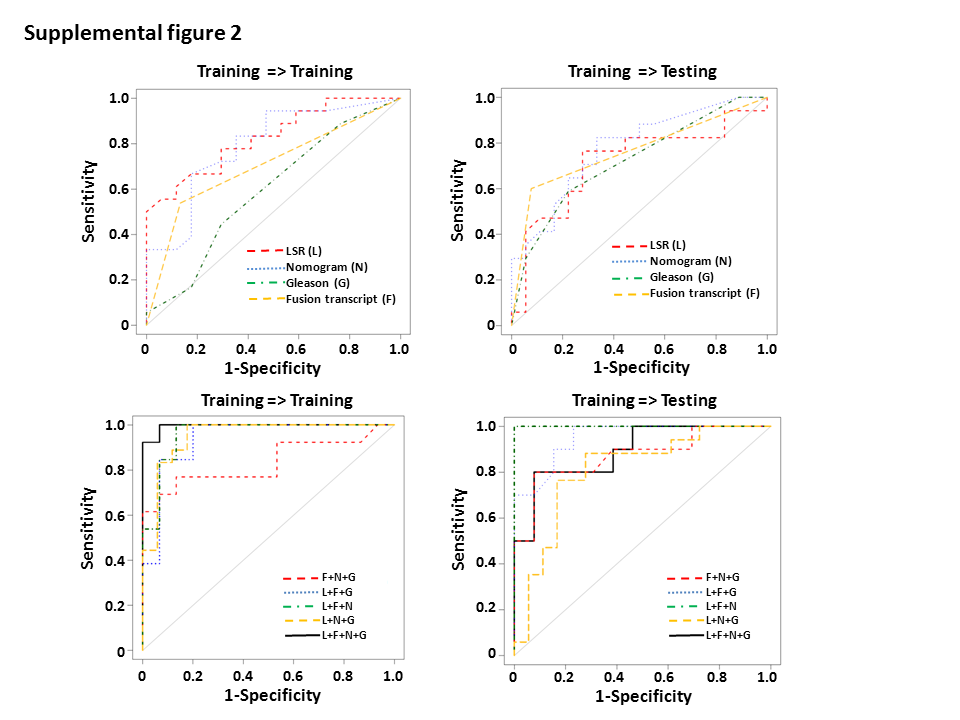

Supplement: S2 Fig — (A) LSR derived from leukocyte genome CNV predicts prostate cancer likely lethality (recurrent within 12 months of radical prostatectomy and PSADT≤4 months). Receiver operating curve (ROC) analyses using LSRs derived from leukocyte CNVs as prediction parameter (red) to predict prostate cancer likely lethality, versus Nomogram (blue), Gleason’s grade (green) and the status of 8 fusion transcripts[14] (yellow). The samples were equally split randomly into training and testing sets 10 times. The ROC analysis represents the results from the most representative split. (B) Combination of LSR (L), Gleason’s grade (G), Nomogram (N) and the status of fusion transcripts (F) to predict prostate cancer likely lethality. ROC analysis of a model combining LSR, fusion transcripts, Nomogram and Gleason’s grade using LDA is indicated by black. ROC analysis of a model combining fusion transcripts, Nomogram and Gleason’s grade using LDA is indicated by red. ROC analysis of a model combining LSR, fusion transcripts and Gleason’s grade using LDA is indicated by blue. ROC analysis of a model combining LSR, fusion transcripts and Nomogram using LDA is indicated by green. ROC analysis of a model combining LSR, Nomogram and Gleason’s grade is indicated by yellow. Similar random splits of training and testing data sets were performed as of (A). (TIF) [file pone.0135982.s002.TIF]

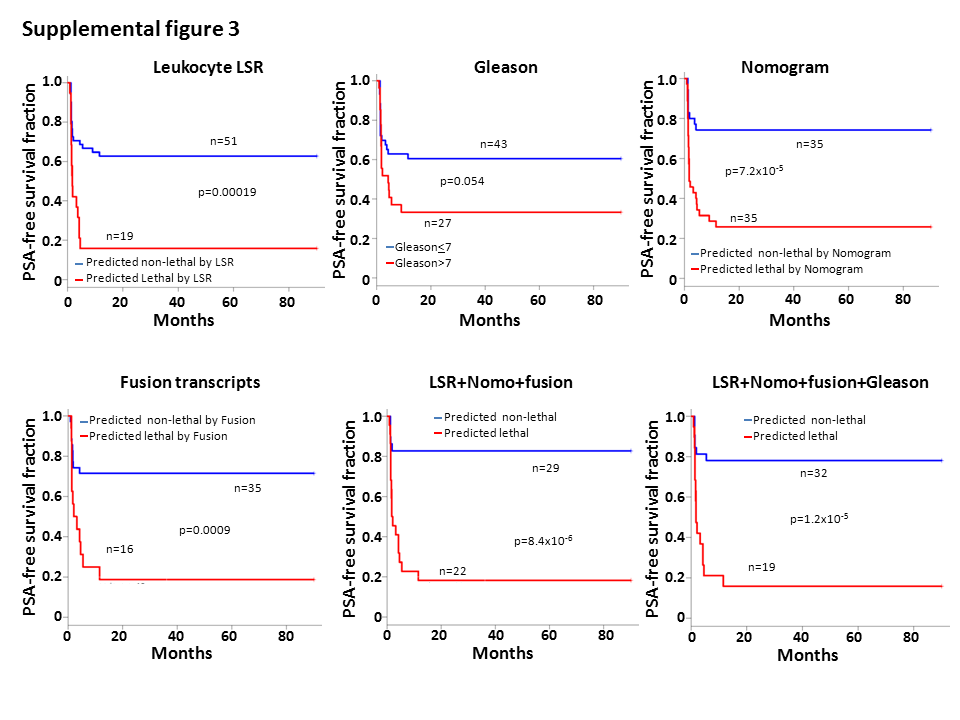

Supplement: S3 Fig — Kaplan-Meier analysis on patients predicted by LSR based on CNV of patients’ leukocytes as likely lethal (recurrent within 12 months of radical prostatectomy and PSADT≤4 months) versus likely non-recurrent (upper left). Similar survival analyses were also performed on case segregations based on Gleason’s grades (upper middle), Nomogram probability (upper right), the status of 8 fusion transcripts (lower left), or a model by combining LSR, Nomogram and fusion transcript status using LDA (lower middle), or a model by combining LSR, Nomogram, Gleason grade and fusion transcript status using LDA (lower right). Number of samples analyzed and p values are indicated. (TIF) [file pone.0135982.s003.TIF]
