# Supplementary material for: Genomic Copy Number Variations in the Genomes of Leukocytes Predict Prostate Cancer Clinical Outcomes
Source: PLoS One. 2015 Aug 21;10(8):e0135982. doi: 10.1371/journal.pone.0135982 (PMC4546524; doi:10.1371/journal.pone.0135982)
Supplement: S1 Table — (DOCX) [file pone.0135982.s004.docx]

**Supplemental Table 1: Clinical information for 143 blood samples**

| Case name | Age | Race | Pre-operative PSA | Gleason grad | Pathological stage | 5-year Nomogram | Prostate cancer recurrence | Fast recurrence | Time to relapse (Month) | PSADT | Surgical year |
| --- | --- | --- | --- | --- | --- | --- | --- | --- | --- | --- | --- |
| 11563B | 70s | W | 8.4 | 3+4=7 | T1cN0MX | 0.97 | no | nf | >90 | N/A | 1998 |
| 1199B | 50s | W | 40 | 3+5=8 | T3bN0MX | 0.88 | yes | nf | 15 | 33.7 | 1999 |
| 13745B | 60s | W | 6.8 | 3+4=7 | T1cN0MX | 0.97 | no | nf | >90 | N/A | 1998 |
| 16464B | 60s | W | 8.5 | 3+4=7 | T3bN0MX | 0.92 | yes | nf | 88.5 | 24.6 | 1999 |
| 18176B | 50s | W | 8.8 | 3+3=6 | T2bN0MX | 0.98 | yes | nf | 87 | 26.9 | 1999 |
| 1942B | 60s | W | 7.5 | 4+5=9 | T3bN0MX | 0.73 | yes | nf | 80.1 | 14.8 | 1998 |
| 25313B | 50s | W | 9.5 | 5+3=8 | T3bN0MX | 0.83 | no | nf | >90 | N/A | 1998 |
| 27086B | 50s | W | 9.5 | 3+3=6 | T2BN0MX | 0.98 | no | nf | >90 | N/A | 1998 |
| 28685B | 50s | W | 56.6 | 4+3=7 | T3AN0MX | 0.75 | yes | nf | 77.5 | 17.7 | 1998 |
| 28685B2 | 50s | W | 50.2 | 4+3=7 | T3AN0MX | 0.76 | yes | nf | 79.6 | 17.7 | 1998 |
| 4308B | 60s | W | 12.4 | 3+3=6 | T1CN0MX | 0.98 | no | nf | >90 | N/A | 1998 |
| 4336B | 60s | W | 2.5 | 3+3=6 | T1cN0MX | 0.99 | yes | nf | 21.7 | 22.0 | 1997 |
| 4851B | 60s | W | 7 | 4+3=7 | T1CN0MX | 0.94 | no | nf | >90 | N/A | 1998 |
| 5396B | 60s | W | 9.1 | 5+4=9 | T2bN1MX | 0.88 | no | nf | >90 | N/A | 2003 |
| 562B | 60s | W | 5.5 | 3+3=6 | T2AN0MX | 0.98 | no | nf | >90 | N/A | 1998 |
| 6634B | 50s | U | 18.2 | 3+3=6 | T2bN0MX | 0.98 | no | nf | >90 | N/A | 1998 |
| 6634B2 | 50s | U | 18.2 | 3+3=6 | T2bN0MX | 0.98 | no | nf | >90 | N/A | 1998 |
| 678B | 70s | W | 10.8 | 4+5=9 | T3bN0MX | 0.71 | no | nf | >90 | N/A | 2000 |
| 7270B | 70s | W | 4.1 | 3+4=7 | T3BN1MX | 0.94 | no | nf | >90 | N/A | 2000 |
| 7504B | 70s | U | 10.5 | 4+5=9 | T3bN0MX | 0.71 | no | nf | >90 | N/A | 1999 |
| 9122B | 50s | W | 13 | 3+4=7 | T1CN0MX | 0.97 | no | nf | >90 | N/A | 1997 |
| 9122B2 | 50s | W | 14.4 | 3+4=7 | T1CN0MX | 0.96 | no | nf | >90 | N/A | 1997 |
| DB237B | 70s | W | 6.3 | 3+3=6 | T2bN0MX | 0.98 | yes | nf | 46 | 25.97 | 2001 |
| DB237B2 | 70s | W | 6.1 | 3+3=6 | T2bN0MX | 0.98 | yes | nf | 42.3 | 26.24 | 2000 |
| FB104 | 60s | W | 16.6 | 4+4=8 | T3b N0 MX | 0.78 | yes | f | 22.5 | 3.2 | 2003 |
| FB120B | 60s | W | 61.1 | 3+4=7 | T3aN0MX | 0.88 | yes | nf | 1.3 | 20.84 | 2003 |
| FB174B | 60s | W | 6.9 | 3+4=7 | T3aN0MX | 0.93 | yes | f | 30.5 | 3.21 | 2003 |
| FB183B | 60s | W | 9.7 | 3+4=7 | T2cN0MX | 0.97 | yes | nf | 78.8 | 25.6 | 2003 |
| FB222B | 50s | W | 25.9 | 4+3=7 | T3a N0 MX | 0.73 | yes | f | 1.2 | 2.4 | 2003 |
| FB238B | 60s | W | 15.9 | 3+4=7 | T3bN0MX | 0.91 | yes | nf | 30 | 29.97 | 2003 |
| FB41B | 60s | AA | 7.9 | 3+4=7 | T2c N0 MX | 0.97 | yes | f | 82.1 | 4.1 | 2003 |
| FB421B | 60s | W | 4.5 | 3+4=7 | T3aN0MX | 0.94 | yes | f | 1.3 | 4.37 | 2003 |
| FB493B | 50s | AA | 7.1 | 3+3=6 | T3aN0MX | 0.96 | yes | nf | 62.5 | 17.84 | 2003 |
| FB586B | 50s | W | 7.2 | 3+4=7 | T3aN0Mx | 0.93 | yes | nf | 46.6 | 15.6 | 2004 |
| FB94B | 60s | W | 12.9 | 3+4=7 | T2cN0MX | 0.97 | yes | nf | 3.4 | 15.16 | 2003 |
| FB95 | 60s | W | 2.9 | 4+5=9 | T3a N0 MX | 0.81 | yes | N/A | 17 | N/A | 2003 |
| GB195B | 60s | W | 10.1 | 3+4=7 | T2cN0MX | 0.97 | yes | nf | 53.2 | 23.8 | 2006 |
| GB222 | 60s | W | 6.8 | 3+3=6 | T2c N0 MX | 0.98 | yes | f | 34.9 | 3.9 | 2004 |
| GB368 | 60s | W | 5.5 | 4+3=7 | T3a N0 MX | 0.86 | yes | nf | 70.1 | 18 | 2004 |
| GB400B | 60s | W | 3.5 | 3+4=7 | T3bN0MX | 0.94 | yes | f | 29.6 | 4.22 | 2005 |
| HB021B | 50s | W | 5.9 | 3+3=6 | T2bN0MX | 0.98 | yes | f | 24.2 | 3.99 | 2004 |
| HB033B | 50s | W | 8.4 | 3+4=7 | T2cN0MX | 0.97 | no | nf | >90 | N/A | 2004 |
| HB207B | 60s | W | 6.3 | 4+5=9 | T3bN0MX | 0.75 | yes | f | 5.5 | 0.58 | 2005 |
| HB235B | 60s | W | 4.6 | 4+5=9 | T3bN1MX | 0.67 | yes | nf | 1.3 | 20.76 | 2010 |
| HB261B | 50s | W | 5.4 | 3+4=7 | T3aN0MX | 0.94 | no | nf | >90 | N/A | 2005 |
| HB303 | 60s | W | 31.3 | 3+4=7 | T2c N0 MX | 0.96 | no | nf | >90 | N/A | 2005 |
| HB305B | 60s | W | 10.1 | 3+3=6 | T3bN0MX | 0.95 | yes | f | 1.4 | 3.9 | 2005 |
| HB312B | 70s | W | 1.1 | 4+4=8 | T3bN0MX | 0.86 | yes | nf | 7.4 | 15.23 | 2005 |
| HB327 | 60s | W | 9.5 | 4+4=8 | T2c N0 MX | 0.88 | no | nf | >90 | N/A | 2005 |
| HB340 | 60s | W | 9.57 | 3+4=7 | T2c N0 MX | 0.97 | yes | N/A | 4.54 | N/A | 2005 |
| HB346 | 60s | W | 17.2 | 3+4=7 | T3a N0 MX | 0.91 | no | nf | >90 | N/A | 2005 |
| HB46B | 60s | W | 4.7 | 4+4=8 | T3bN0MX | 0.77 | yes | nf | 20.1 | 15.28 | 2005 |
| HB492 | 60s | W | 7.4 | 3+4=7 | T2c N0 MX | 0.97 | yes | nf | 82.3 | 24 | 2005 |
| HB504B | 50s | U | 70 | 4+4=8 | T3bN0MX | 0.57 | yes | f | 4.3 | 0.69 | 2006 |
| HB526B | 60s | W | 8.7 | 3+3=6 | T3bN0MX | 0.95 | yes | f | 1.4 | 2.66 | 2009 |
| HB568B | 60s | W | 4.4 | 3+4=7 | T3bN0MX | 0.94 | yes | f | 22.4 | 4.19 | 2005 |
| HB591B | 60s | W | 13.6 | 3+4=7 | T3bN1MX | 0.87 | yes | f | 1.3 | 4.48 | 2007 |
| HB603B | 60s | W | 8.4 | 3+4=7 | T3aN1MX | 0.89 | yes | f | 22.1 | 11.91 | 2005 |
| HB658 | 60s | W | 20.6 | 4+3=7 | T3b N0 MX | 0.79 | no | nf | >90 | N/A | 2005 |
| HB705 | 60s | W | 9.8 | 4+3=7 | T2c N0 MX | 0.93 | no | nf | >90 | N/A | 2005 |
| IB071B | 60s | W | 2.6 | 3+4=7 | T3aN0MX | 0.95 | yes | f | 4.3 | 1.58 | 2007 |
| IB111 | 60s | W | 9.5 | 3+4=7 | T2c N0 MX | 0.97 | no | nf | >90 | N/A | 2006 |
| IB112B | 60s | U | 4.7 | 3+4=7 | T3aN0MX | 0.94 | yes | nf | 55.8 | 30.59 | 2006 |
| IB113B | 70s | W | 5.6 | 3+4=7 | T3bN0MX | 0.93 | yes | nf | 47.3 | 20.62 | 2005 |
| IB133 | 60s | W | 4.6 | 3+4=7 | T2c N0 MX | 0.97 | yes | N/A | 34.9 | N/A | 2005 |
| IB134B | 70s | W | 15.7 | 4+5=9 | T3bN0MX | 0.68 | no | nf | >90 | N/A | 2005 |
| IB135 | 60s | W | 31.9 | 4+3=7 | T3b N1 MX | 0.67 | yes | f | 35.2 | 2.2 | 2006 |
| IB136B | 50s | W | 19.6 | 4+4=8 | T3bN1MX | 0.54 | yes | f | 1.8 | 2.23 | 2005 |
| IB180 | 60s | W | 3 | 3+4=7 | T2c N0 MX | 0.98 | no | nf | >90 | N/A | 2006 |
| IB289 | 60s | W | 9.96 | 3+4=7 | T2a N0 MX | 0.97 | no | nf | >90 | N/A | 2006 |
| IB298B | 60s | W | 5.3 | 3+4=7 | T3bN0MX | 0.93 | yes | nf | 34.3 | 20.4 | 2006 |
| IB378 | 60s | W | 2.8 | 4+3=7 | T3b N0 MX | 0.88 | no | nf | >90 | N/A | 2006 |
| IB483B | 50s | W | 5.2 | 3+4=7 | T2bN0MX | 0.97 | yes | f | 1.4 | 1.7 | 2007 |
| JB608 | 60s | W | 6.76 | 3+4=7 | T3a N0 MX | 0.93 | yes | f | 1.3 | 0.6 | 2007 |
| IB627 | 60s | W | 7.86 | 3+4=7 | T2c N0 MX | 0.97 | yes | N/A | 10.5 | N/A | 2006 |
| IB673 | 60s | W | 5.7 | 4+4=8 | T3a N0 MX | 0.77 | yes | N/A | 22.8 | N/A | 2006 |
| IB684B | 60s | W | 4.1 | 3+4=7 | T3bN0MX | 0.94 | yes | nf | 60.9 | 77.4 | 2006 |
| JB378B | 60s | W | 5 | 3+3=6 | T2bN0MX | 0.99 | yes | nf | 18.4 | 45.8 | 2008 |
| JB426B | 60s | W | 5.7 | 3+4=7 | T2cN0MX | 0.97 | yes | f | 17.4 | 2.26 | 2007 |
| JB770B | 60s | W | 2.4 | 4+4=8 | T2cN0MX | 0.92 | yes | f | 33.8 | 2.99 | 2008 |
| KB170B | 70s | W | 14.1 | 3+4=7 | T3bN1MX | 0.87 | yes | f | 1.8 | 4.22 | 2008 |
| PR018B | 60s | W | 9 | 3+4=7 | T3aN0MX | 0.93 | yes | nf | 78 | 55.02 | 1999 |
| PR048 | 60s | W | 5.9 | 4+3=7 | T3a N0 MX | 0.86 | no | nf | >90 | N/A | 2002 |
| PR065 | 60s | W | 10.2 | 4+5=9 | T4 N0 MX | 0.88 | yes | f | 16.7 | 2.1 | 2001 |
| PR073 | 60s | W | 7.8 | 3+5=8 | T3a N0 MX | 0.93 | yes | f | 36.6 | 0.2 | 2000 |
| PR079B | 60s | W | 5.1 | 3+4=7 | T3aN0MX | 0.94 | yes | nf | 85.3 | 17.32 | 2000 |
| PR150 | 60s | W | 14.98 | 3+4=7 | T2b N0 MX | 0.96 | yes | N/A | 36.1 | N/A | 2001 |
| PR151B | 60s | W | 8.1 | 4+3=7 | T2bN0MX | 0.93 | yes | nf | 35.5 | 35.19 | 2001 |
| PR151B2 | 60s | W | 8.9 | 4+3=7 | T2bN0MX | 0.93 | yes | nf | 36.9 | 26.65 | 2001 |
| PR227 | 60s | W | 4.46 | 3+4=7 | T2c N0 MX | 0.97 | no | nf | >90 | N/A | 2002 |
| PR236B | 60s | W | 9.9 | 5+5=10 | T3bN0MX | 0.71 | yes | f | 1.3 | 3.91 | 2006 |
| PR300B | 50s | W | 20.3 | 3+4=7 | T3bN1MX | 0.85 | yes | f | 59 | 3.87 | 2003 |
| PR303B | 70s | W | 10.5 | 3+3=6 | T3bN0MX | 0.95 | yes | nf | 54.6 | 43.29 | 2004 |
| PR304B | 60s | W | 5.9 | 4+4=8 | T3bN0MX | 0.75 | yes | nf | 47.4 | 32.75 | 2002 |
| PR306B | 60s | W | 11.5 | 3+4=7 | T3bN0MX | 0.92 | yes | nf | 16.4 | 52.93 | 2002 |
| PR310B | 60s | W | 5.1 | 3+4=7 | T3bN0MX | 0.93 | yes | f | 22.8 | 1.58 | 2007 |
| PR311B | 60s | W | 10.2 | 4+4=8 | T3bN0MX | 0.71 | yes | nf | 61.6 | 160 | 2002 |
| PR363B | 60s | W | 12.5 | 3+4=7 | T2bN0Mx | 0.97 | yes | nf | 54 | 26 | 2002 |
| PR372 | 60s | W | 11.2 | 4+4=8 | T3a N0 MX | 0.72 | yes | f | 4.5 | 1.4 | 2001 |
| PR375B | 50s | W | 11.3 | 3+4=7 | T3bN1MX | 0.87 | yes | f | 1.2 | 1.13 | 2002 |
| PR434B | 60s | W | 6.4 | 3+4=7 | T3aN0MX | 0.93 | yes | nf | 72.8 | 30.81 | 2000 |
| PR485 | 60s | W | 7.7 | 3+4=7 | T2b N0 MX | 0.97 | yes | f | 35.2 | 2.1 | 2001 |
| PR490B | 60s | W | 5.7 | 3+4=7 | T2AN0MX | 0.97 | yes | nf | 45.5 | 35.6 | 1999 |
| PR521B | 50s | W | 6.4 | 3+4=7 | T2bN0MX | 0.97 | yes | nf | 79.2 | 15.51 | 2001 |
| PR524 | 60s | W | 8.5 | 3+2=5 | T2b N0 MX | 0.98 | yes | N/A | 1.6 | N/A | 2000 |
| PR525 | 60s | W | 6.3 | 3+3=6 | T2a N0 MX | 0.98 | yes | N/A | 18.4 | N/A | 2000 |
| PR527 | 60s | AA | 9.1 | 3+4=7 | T2b N0 MX | 0.97 | yes | f | 3.78 | 3.78 | 2001 |
| PR528 | 60s | W | 1.3 | 3+3=6 | T3a N0 MX | 0.98 | yes | N/A | 36.8 | N/A | 2000 |
| PR529 | 60s | W | 6.7 | 3+4=7 | T2b N0 MX | 0.97 | yes | N/A | 16.6 | N/A | 2002 |
| PR530 | 60s | W | 4.4 | 3+4=7 | T2c N0 MX | 0.98 | yes | N/A | 30 | N/A | 2002 |
| PR535 | 60s | W | 7 | 3+4=7 | T2b N0 MX | 0.97 | no | nf | >90 | N/A | 2000 |
| PR536 | 60s | W | 5.4 | 3+4=7 | T2b N0 MX | 0.97 | no | nf | >90 | N/A | 2002 |
| PR537 | 60s | W | 5.4 | 3+3=6 | T2b N0 MX | 0.98 | no | nf | >90 | N/A | 2001 |
| PR541 | 60s | W | 29.4 | 4+4=8 | T3b N0 MX | 0.64 | no | nf | >90 | N/A | 2002 |
| PR542 | 60s | W | 11.6 | 4+4=8 | T3b N0 MX | 0.7 | no | nf | >90 | N/A | 2000 |
| PR543 | 60s | W | 20.8 | 4+4=8 | T3a N0 MX | 0.68 | no | nf | >90 | N/A | 2000 |
| TP08-S00262 | 60s | W | 22.8 | 4+5=9 | T3b N0 MX | 0.66 | yes | f | 1.6 | 0.2 | 2008 |
| TP08-S00268B | 60s | W | 2 | 3+4=7 | T2bN0MX | 0.98 | yes | f | 21.4 | 3.8 | 2009 |
| TP08-S00530B | 60s | W | 11.1 | 3+4=7 | T3bN0MX | 0.92 | yes | f | 1.3 | 3.31 | 2008 |
| TP08-S00542B | 50s | W | 4.3 | 3+4=7 | T2cN0MX | 0.98 | yes | f | 1.9 | 3.61 | 2009 |
| TP09-S0006B | 50s | W | 4.9 | 4+4=8 | T3bN1MX | 0.66 | yes | f | 4.6 | 1.23 | 2009 |
| TP09-S0408B | 70s | U | 2.9 | 4+4=8 | T3aN0MX | 0.81 | yes | f | 1.5 | 3.18 | 2010 |
| TP09-S0420B | 50s | W | 14.6 | 3+4=7 | T3bN1MX | 0.86 | yes | f | 1.4 | 3.7 | 2009 |
| TP09-S0420B2 | 50s | W | 12.8 | 3+4=7 | T3bN1MX | 0.87 | yes | f | 3.2 | 2.6 | 2009 |
| TP09-S0638B | 50s | W | 9.2 | 3+4=7 | T3bN1MX | 0.88 | yes | f | 1.4 | 1.83 | 1999 |
| TP09-S0721B | 50s | W | 29.3 | 3+4=7 | T3bN1MX | 0.84 | yes | f | 1.4 | 0.93 | 2010 |
| TP09-S0928 | 60s | W | 5.9 | 4+4=8 | T3b N1 MX | 0.64 | yes | f | 1.3 | 0.1 | 2012 |
| TP10-S093B | 60s | W | 4.1 | 3+4=7 | T3aN0MX | 0.94 | yes | nf | 43.8 | 39.96 | 2000 |
| TP12-S0740 | 50s | W | 25 | 4+5=9 | T3b N0 MX | 0.65 | yes | f | 1.6 | 0.4 | 2012 |
| TP12-S0786 | 60s | W | 4.5 | 4+3=7 | T3b N1 MX | 0.8 | yes | f | 1.2 | 0.6 | 2012 |
| TP12-S0790 | 60s | W | 24.2 | 4+3=7 | T3a N0 MX | 0.8 | yes | f | 11.6 | 3.7 | 2012 |
| TP12-S0799 | 50s | W | 6.4 | 4+3=7 | T3a N0 MX | 0.86 | yes | N/A | 13.1 | N/A | 2012 |
| TP12-S0805 | 50s | W | 7.6 | 4+3=7 | T3a N1 MX | 0.78 | yes | N/A | 6.18 | N/A | 2013 |
| TP12-S0918 | 60s | W | 6.8 | 5+4=9 | T3b N1 MX | 0.63 | yes | f | 0.9 | 2.3 | 2012 |
| TP12-S0945 | 50s | W | 10.3 | 4+5=9 | T3a N1 MX | 0.61 | yes | f | 9.1 | 3.1 | 2012 |
| TP12-S0996 | 60s | W | 6.3 | 4+3=7 | T3a N1 MX | 0.79 | yes | f | 1.4 | 0.4 | 2012 |
| TP12-S1059 | 60s | W | 10.6 | 4+4=8 | T3a N0 MX | 0.73 | yes | f | 1.3 | 0.43 | 2012 |
| TP12-S1303 | 60s | W | 9.87 | 4+5=9 | T3b N1 MX | 0.6 | yes | f | 1.78 | 0.5 | 2012 |
| TP13-S0048 | 60s | W | 22 | 4+4=8 | T3a N0 MX | 0.68 | yes | f | 1.54 | 4 | 2012 |
| TP13-S0109 | 60s | W | 21.46 | 4+4=8 | T3b N1 MX | 0.53 | yes | f | 1.7 | 1.4 | 2013 |
| TP13-S0147 | 60s | W | 14.1 | 4+3=7 | T2c N0 MX | 0.92 | yes | N/A | 5.4 | N/A | 2013 |
| TP13-S0248 | 60s | W | 6.8 | 4+4=8 | T3b N1 MX | 0.63 | yes | f | 2.1 | 0.52 | 2013 |
| TP13-S0456 | 50s | W | 29.9 | 4+5=9 | T3a N0 MX | 0.66 | yes | f | 1.8 | 1.87 | 2013 |
