# Supplementary material for: Genomic Copy Number Variations in the Genomes of Leukocytes Predict Prostate Cancer Clinical Outcomes
Source: PLoS One. 2015 Aug 21;10(8):e0135982. doi: 10.1371/journal.pone.0135982 (PMC4546524; doi:10.1371/journal.pone.0135982)
Supplement: S2 Table — (DOCX) [file pone.0135982.s005.docx]

| **Supplemental Table 2: Prediction of prostate cancer recurrence based on leukocyte LSR, Gleason, Nomogram and fusion transcript status (the representative result for Figure 3)** | | | | | | |
| --- | --- | --- | --- | --- | --- | --- |
|  |  |  |  |  |  |  |
| **Model** | **Accuracy** | **Sensitivity** | **Specificity** | **Youden index** | **AUC** | **ROC p-value** |
| **Equal split training data (n=72)** | | | | | | |
| LSR | 0.778 | 0.8 | 0.706 | 0.506 | 0.775 | 1.52 x 10^-4^ |
| Nomogram | 0.681 | 0.691 | 0.647 | 0.338 | 0.619 | 1.43 x 10^-1^ |
| Gleason | 0.347 | 0.218 | 0.765 | -0.017 | 0.496 | 9.54 x 10^-1^ |
| Fusion | 0.651 | 0.586 | 0.786 | 0.372 | 0.686 | 1.53 x 10^-2^ |
| L+N+F | 0.837 | 0.793 | 0.929 | 0.722 | 0.897 | 2.60 x 10^-9^ |
| L+N+G | 0.639 | 0.545 | 0.941 | 0.487 | 0.778 | 7.34 x 10^-5^ |
| N+F+G | 0.721 | 0.586 | 1 | 0.586 | 0.787 | 1.39 x 10^-4^ |
| L+F+G | 0.814 | 0.759 | 0.929 | 0.687 | 0.897 | 5.44 x 10^-9^ |
| L+N+F+G | 0.86 | 0.897 | 0.786 | 0.682 | 0.906 | 1.99 x 10^-9^ |
|  |  |  |  |  |  |  |
| **Equal split testing data (n=71)** | | | | | | |
| LSR | 0.761 | 0.792 | 0.667 | 0.459 | 0.768 | 8.10 x 10^-5^ |
| Nomogram | 0.648 | 0.736 | 0.389 | 0.125 | 0.596 | 2.06 x 10^-1^ |
| Gleason | 0.451 | 0.358 | 0.722 | 0.081 | 0.558 | 4.33 x 10^-1^ |
| Fusion | 0.638 | 0.485 | 1 | 0.485 | 0.742 | 1.68 x 10^-6^ |
| L+N+F | 0.872 | 0.909 | 0.786 | 0.695 | 0.898 | 1.13 x 10^-9^ |
| L+N+G | 0.634 | 0.604 | 0.722 | 0.326 | 0.761 | 3.68 x 10^-4^ |
| N+F+G | 0.596 | 0.424 | 1 | 0.424 | 0.714 | 5.89 x 10^-3^ |
| L+F+G | 0.745 | 0.727 | 0.786 | 0.513 | 0.89 | 2.68 x 10^-9^ |
| L+N+F+G | 0.851 | 0.909 | 0.714 | 0.623 | 0.892 | 1.34 x 10^-9^ |

L-LSR; N-Nomogram; F-fusion transcript status; G-Gleason grade;

L+N+F: LDA model to combine LSR, Nomogram and fusion transcript status;

L+N+G: LDA model to combine LSR, Nomogram and Gleason grade;

N+F+G: LDA model to combine Nomogram, fusion transcript status and Gleason grade;

L+N+F+G: LDA model to combine LSR, Nomogram, fusion transcript status and Gleason grade.
