# Supplementary material for: Genomic Copy Number Variations in the Genomes of Leukocytes Predict Prostate Cancer Clinical Outcomes
Source: PLoS One. 2015 Aug 21;10(8):e0135982. doi: 10.1371/journal.pone.0135982 (PMC4546524; doi:10.1371/journal.pone.0135982)
Supplement: S3 Table — (DOCX) [file pone.0135982.s006.docx]

**Supplemental Table 3: Pairwise ROC p-value for prostate cancer recurrent status prediction (the geometric mean of the 10 cross-validations)**

**Training => Training**

|  | LSR | Nomogram | Gleason | Fusion | L+F+N+G | F+N+G | L+F+G | L+F+N | L+N+G |
| --- | --- | --- | --- | --- | --- | --- | --- | --- | --- |
| LSR | 1 | 8.09E-2 | 8.63E-3 | 4.56E-1 | 5.38E-2 | 5.96E-1 | 8.84E-2 | 6.73E-2 | 4.07E-1 |
| Nomogram |  | 1 | 2.79E-2 | 2.73E-1 | 7.47E-4 | 8.87E-2 | 1.62E-3 | 1.07E-3 | 3.37E-2 |
| Gleason |  |  | 1 | 5.52E-2 | 1.18E-5 | 7.73E-3 | 3.40E-5 | 1.83E-5 | 2.65E-3 |
| Fusion |  |  |  | 1 | 1.50E-3 | 9.60E-2 | 2.68E-3 | 1.94E-3 | 2.77E-1 |
| L+F+N+G |  |  |  |  | 1 | 4.41E-2 | 5.15E-1 | 5.42E-1 | 1.09E-1 |
| F+N+G |  |  |  |  |  | 1 | 8.49E-2 | 6.62E-2 | 6.84E-1 |
| L+F+G |  |  |  |  |  |  | 1 | 6.53E-1 | 1.61E-1 |
| L+F+N |  |  |  |  |  |  |  | 1 | 1.33E-1 |
| L+N+G |  |  |  |  |  |  |  |  | 1 |

L-LSR; N-Nomogram; F-fusion transcript status; G-Gleason grade;

L+N+F: LDA model to combine LSR, Nomogram and fusion transcript status;

L+N+G: LDA model to combine LSR, Nomogram and Gleason grade;

N+F+G: LDA model to combine Nomogram, fusion transcript status and Gleason grade;

L+N+F+G: LDA model to combine LSR, Nomogram, fusion transcript status and Gleason grade.

**Training => Testing**

|  | LSR | Nomogram | Gleason | Fusion | L+F+N+G | F+N+G | L+F+G | L+F+N | L+N+G |
| --- | --- | --- | --- | --- | --- | --- | --- | --- | --- |
| LSR | 1 | 7.28E-2 | 5.78E-3 | 5.15E-1 | 2.86E-1 | 4.61E-1 | 2.57E-1 | 1.58E-1 | 2.52E-1 |
| Nomogram |  | 1 | 1.51E-1 | 1.87E-1 | 6.84E-3 | 2.37E-1 | 5.94E-3 | 2.10E-3 | 1.65E-1 |
| Gleason |  |  | 1 | 2.90E-2 | 3.21E-4 | 5.01E-2 | 2.64E-4 | 5.73E-5 | 3.33E-3 |
| Fusion |  |  |  | 1 | 2.51E-2 | 4.47E-1 | 1.55E-2 | 8.23E-3 | 6.08E-1 |
| L+F+N+G |  |  |  |  | 1 | 3.71E-2 | 3.56E-1 | 2.56E-1 | 1.59E-1 |
| F+N+G |  |  |  |  |  | 1 | 4.51E-2 | 2.06E-2 | 6.07E-1 |
| L+F+G |  |  |  |  |  |  | 1 | 1.77E-1 | 1.37E-1 |
| L+F+N |  |  |  |  |  |  |  | 1 | 7.87E-2 |
| L+N+G |  |  |  |  |  |  |  |  | 1 |

L-LSR; N-Nomogram; F-fusion transcript status; G-Gleason grade;

L+N+F: LDA model to combine LSR, Nomogram and fusion transcript status;

L+N+G: LDA model to combine LSR, Nomogram and Gleason grade;

N+F+G: LDA model to combine Nomogram, fusion transcript status and Gleason grade;

L+N+F+G: LDA model to combine LSR, Nomogram, fusion transcript status and Gleason grade.
