# Supplementary material for: Genomic Copy Number Variations in the Genomes of Leukocytes Predict Prostate Cancer Clinical Outcomes
Source: PLoS One. 2015 Aug 21;10(8):e0135982. doi: 10.1371/journal.pone.0135982 (PMC4546524; doi:10.1371/journal.pone.0135982)
Supplement: S5 Table — (DOCX) [file pone.0135982.s008.docx]

**Supplemental Table 5: Pairwise survival p-value for prostate cancer recurrent status prediction (the geometric mean of the 10 cross-validations)**

|  | LSR | Nomogram | Gleason | Fusion | L+F+N+G | F+N+G | L+F+G | L+F+N | L+N+G |
| --- | --- | --- | --- | --- | --- | --- | --- | --- | --- |
| LSR | 1 | 9.61E-3 | 5.29E-4 | 8.02E-2 | 3.63E-2 | 1.26E-2 | 9.07E-2 | 6.58E-3 | 8.47E-2 |
| Nomogram |  | 1 | 2.14E-2 | 8.48E-3 | 1.25E-2 | 1.42E-2 | 6.00E-3 | 3.45E-4 | 3.67E-2 |
| Gleason |  |  | 1 | 3.54E-4 | 4.98E-4 | 1.64E-3 | 2.52E-4 | 1.82E-5 | 1.44E-3 |
| Fusion |  |  |  | 1 | 7.46E-2 | 2.19E-2 | 8.70E-2 | 1.69E-2 | 7.18E-2 |
| L+F+N+G |  |  |  |  | 1 | 5.70E-2 | 1.37E-1 | 1.21E-2 | 3.47E-2 |
| F+N+G |  |  |  |  |  | 1 | 2.78E-2 | 2.40E-3 | 2.20E-2 |
| L+F+G |  |  |  |  |  |  | 1 | 2.13E-2 | 3.95E-2 |
| L+F+N |  |  |  |  |  |  |  | 1 | 3.18E-3 |
| L+N+G |  |  |  |  |  |  |  |  | 1 |

L-LSR; N-Nomogram; F-fusion transcript status; G-Gleason grade;

L+N+F: LDA model to combine LSR, Nomogram and fusion transcript status;

L+N+G: LDA model to combine LSR, Nomogram and Gleason grade;

N+F+G: LDA model to combine Nomogram, fusion transcript status and Gleason grade;

L+N+F+G: LDA model to combine LSR, Nomogram, fusion transcript status and Gleason grade.
