# Supplementary material for: Genomic Copy Number Variations in the Genomes of Leukocytes Predict Prostate Cancer Clinical Outcomes
Source: PLoS One. 2015 Aug 21;10(8):e0135982. doi: 10.1371/journal.pone.0135982 (PMC4546524; doi:10.1371/journal.pone.0135982)
Supplement: S6 Table — (DOCX) [file pone.0135982.s009.docx]

| **Supplemental Table 6: Prediction of prostate cancer recurrent PSADT<4 months based on leukocyte LSR, Gleason, Nomogram and fusion transcript status (the representative result for Figure 5).** | | | | | | |
| --- | --- | --- | --- | --- | --- | --- |
|  |  |  |  |  |  |  |
| **Model** | **Accuracy** | **Sensitivity** | **Specificity** | **Youden index** | **AUC** | **ROC p-value** |
| **Equal split training data (n=65)** | | | | | | |
| LSR | 0.662 | 0.5 | 0.784 | 0.284 | 0.674 | 1.33 x 10^-2^ |
| Nomogram | 0.677 | 0.536 | 0.784 | 0.319 | 0.668 | 1.65 x 10^-2^ |
| Gleason | 0.415 | 0.292 | 0.765 | 0.056 | 0.555 | 4.69 x 10^-1^ |
| Fusion | 0.667 | 0.579 | 0.731 | 0.31 | 0.655 | 4.00 x 10^-2^ |
| L+N+F | 0.822 | 0.842 | 0.808 | 0.65 | 0.858 | 1.26 x 10^-7^ |
| L+N+G | 0.754 | 0.75 | 0.757 | 0.507 | 0.766 | 4.86 x 10^-5^ |
| N+F+G | 0.8 | 0.632 | 0.923 | 0.555 | 0.764 | 1.99 x 10^-3^ |
| L+F+G | 0.867 | 0.842 | 0.885 | 0.727 | 0.857 | 6.91 x 10^-7^ |
| L+N+F+G | 0.8 | 0.842 | 0.769 | 0.611 | 0.864 | 2.39 x 10^-8^ |
|  |  |  |  |  |  |  |
| **Equal split testing data (n=64)** | | | | | | |
| LSR | 0.547 | 0.259 | 0.757 | 0.016 | 0.65 | 3.49 x 10^-2^ |
| Nomogram | 0.672 | 0.593 | 0.73 | 0.322 | 0.716 | 7.66 x 10^-4^ |
| Gleason | 0.453 | 0.333 | 0.737 | 0.07 | 0.53 | 6.74 x 10^-1^ |
| Fusion | 0.707 | 0.667 | 0.731 | 0.397 | 0.699 | 1.37 x 10^-2^ |
| L+N+F | 0.707 | 0.733 | 0.692 | 0.426 | 0.782 | 3.16 x 10^-4^ |
| L+N+G | 0.656 | 0.593 | 0.703 | 0.295 | 0.727 | 6.57 x 10^-4^ |
| N+F+G | 0.707 | 0.533 | 0.808 | 0.341 | 0.801 | 8.37 x 10^-5^ |
| L+F+G | 0.61 | 0.4 | 0.731 | 0.131 | 0.717 | 9.56 x 10^-3^ |
| L+N+F+G | 0.707 | 0.733 | 0.692 | 0.426 | 0.785 | 2.52 x 10^-4^ |

L-LSR; N-Nomogram; F-fusion transcript status; G-Gleason grade;

L+N+F: LDA model to combine LSR, Nomogram and fusion transcript status;

L+N+G: LDA model to combine LSR, Nomogram and Gleason grade;

N+F+G: LDA model to combine Nomogram, fusion transcript status and Gleason grade;

L+N+F+G: LDA model to combine LSR, Nomogram, fusion transcript status and Gleason grade.
