# Supplementary material for: Genomic Copy Number Variations in the Genomes of Leukocytes Predict Prostate Cancer Clinical Outcomes
Source: PLoS One. 2015 Aug 21;10(8):e0135982. doi: 10.1371/journal.pone.0135982 (PMC4546524; doi:10.1371/journal.pone.0135982)
Supplement: S7 Table — (DOCX) [file pone.0135982.s010.docx]

**Supplemental Table 7: Pairwise ROC p-value for prostate cancer fast-recurrent status prediction (the geometric mean of the 10 cross-validations)**

**Training => Training**

|  | LSR | Nomogram | Gleason | Fusion | L+F+N+G | F+N+G | L+F+G | L+F+N | L+N+G |
| --- | --- | --- | --- | --- | --- | --- | --- | --- | --- |
| LSR | 1 | 8.04E-1 | 1.80E-1 | 5.71E-1 | 2.81E-2 | 2.35E-1 | 6.94E-2 | 3.22E-2 | 2.73E-1 |
| Nomogram |  | 1 | 1.89E-1 | 5.44E-1 | 1.99E-2 | 1.98E-1 | 5.59E-2 | 2.25E-2 | 1.58E-1 |
| Gleason |  |  | 1 | 2.08E-1 | 6.26E-4 | 1.92E-2 | 2.46E-3 | 7.82E-4 | 4.44E-2 |
| Fusion |  |  |  | 1 | 5.93E-3 | 7.92E-2 | 1.00E-2 | 6.61E-3 | 3.90E-1 |
| L+F+N+G |  |  |  |  | 1 | 2.07E-1 | 4.63E-1 | 7.23E-1 | 1.13E-1 |
| F+N+G |  |  |  |  |  | 1 | 4.55E-1 | 2.50E-1 | 4.90E-1 |
| L+F+G |  |  |  |  |  |  | 1 | 4.69E-1 | 2.26E-1 |
| L+F+N |  |  |  |  |  |  |  | 1 | 1.24E-1 |
| L+N+G |  |  |  |  |  |  |  |  | 1 |

L-LSR; N-Nomogram; F-fusion transcript status; G-Gleason grade;

L+N+F: LDA model to combine LSR, Nomogram and fusion transcript status;

L+N+G: LDA model to combine LSR, Nomogram and Gleason grade;

N+F+G: LDA model to combine Nomogram, fusion transcript status and Gleason grade;

L+N+F+G: LDA model to combine LSR, Nomogram, fusion transcript status and Gleason grade.

**Training => Testing**

|  | LSR | Nomogram | Gleason | Fusion | L+F+N+G | F+N+G | L+F+G | L+F+N | L+N+G |
| --- | --- | --- | --- | --- | --- | --- | --- | --- | --- |
| LSR | 1 | 3.76E-1 | 2.99E-1 | 5.36E-1 | 1.10E-1 | 1.68E-1 | 1.61E-1 | 8.17E-2 | 1.36E-1 |
| Nomogram |  | 1 | 6.40E-2 | 5.25E-1 | 3.68E-1 | 4.38E-1 | 4.69E-1 | 2.93E-1 | 6.03E-1 |
| Gleason |  |  | 1 | 1.61E-1 | 1.07E-2 | 2.15E-2 | 2.10E-2 | 7.48E-3 | 5.90E-2 |
| Fusion |  |  |  | 1 | 1.01E-1 | 1.29E-1 | 9.96E-2 | 6.30E-2 | 5.55E-1 |
| L+F+N+G |  |  |  |  | 1 | 2.48E-1 | 3.95E-1 | 3.52E-1 | 4.32E-1 |
| F+N+G |  |  |  |  |  | 1 | 5.11E-1 | 2.31E-1 | 5.50E-1 |
| L+F+G |  |  |  |  |  |  | 1 | 3.85E-1 | 5.49E-1 |
| L+F+N |  |  |  |  |  |  |  | 1 | 3.61E-1 |
| L+N+G |  |  |  |  |  |  |  |  | 1 |

L-LSR; N-Nomogram; F-fusion transcript status; G-Gleason grade;

L+N+F: LDA model to combine LSR, Nomogram and fusion transcript status;

L+N+G: LDA model to combine LSR, Nomogram and Gleason grade;

N+F+G: LDA model to combine Nomogram, fusion transcript status and Gleason grade;

L+N+F+G: LDA model to combine LSR, Nomogram, fusion transcript status and Gleason grade.
