# Supplementary material for: Genomic Copy Number Variations in the Genomes of Leukocytes Predict Prostate Cancer Clinical Outcomes
Source: PLoS One. 2015 Aug 21;10(8):e0135982. doi: 10.1371/journal.pone.0135982 (PMC4546524; doi:10.1371/journal.pone.0135982)
Supplement: S9 Table — (DOCX) [file pone.0135982.s012.docx]

**Supplemental Table 9: Pairwise survival p-value for prostate cancer fast-recurrent status prediction (the geometric mean of the 10 cross-validations)**

|  | LSR | Nomogram | Gleason | Fusion | L+F+N+G | F+N+G | L+F+G | L+F+N | L+N+G |
| --- | --- | --- | --- | --- | --- | --- | --- | --- | --- |
| LSR | 1 | 5.73E-2 | 3.05E-2 | 4.23E-3 | 1.93E-5 | 3.52E-4 | 3.11E-4 | 1.77E-4 | 3.74E-3 |
| Nomogram |  | 1 | 1.59E-2 | 7.73E-3 | 3.03E-5 | 5.88E-4 | 7.40E-4 | 2.99E-4 | 9.52E-3 |
| Gleason |  |  | 1 | 2.41E-4 | 1.50E-6 | 2.09E-5 | 2.75E-5 | 1.16E-5 | 3.68E-4 |
| Fusion |  |  |  | 1 | 1.83E-3 | 1.75E-2 | 9.05E-3 | 2.16E-2 | 7.07E-2 |
| L+F+N+G |  |  |  |  | 1 | 8.64E-3 | 9.32E-3 | 4.50E-2 | 7.81E-4 |
| F+N+G |  |  |  |  |  | 1 | 7.12E-3 | 2.55E-2 | 7.03E-3 |
| L+F+G |  |  |  |  |  |  | 1 | 7.05E-3 | 4.54E-3 |
| L+F+N |  |  |  |  |  |  |  | 1 | 8.65E-3 |
| L+N+G |  |  |  |  |  |  |  |  | 1 |

L-LSR; N-Nomogram; F-fusion transcript status; G-Gleason grade;

L+N+F: LDA model to combine LSR, Nomogram and fusion transcript status;

L+N+G: LDA model to combine LSR, Nomogram and Gleason grade;

N+F+G: LDA model to combine Nomogram, fusion transcript status and Gleason grade;

L+N+F+G: LDA model to combine LSR, Nomogram, fusion transcript status and Gleason grade.
