# Supplementary material for: Genomic Copy Number Variations in the Genomes of Leukocytes Predict Prostate Cancer Clinical Outcomes
Source: PLoS One. 2015 Aug 21;10(8):e0135982. doi: 10.1371/journal.pone.0135982 (PMC4546524; doi:10.1371/journal.pone.0135982)
Supplement: S10 Table — (DOCX) [file pone.0135982.s013.docx]

| **Supplemental Table 10: Prediction of lethal prostate cancer recurrent (PSADT<4 months and relapse time <12 months) VS non-recurrence based on leukocyte LSR, Gleason, Nomogram and fusion transcript status (the average result).** | | | | | | |
| --- | --- | --- | --- | --- | --- | --- |
|  |  |  |  |  |  |  |
| **Model** | **Accuracy** | **Sensitivity** | **Specificity** | **Youden index** | **AUC** | **ROC p-value** |
| **Equal split training data (n=35)** | | | | | | |
| LSR | 0.783 | 0.739 | 0.829 | 0.568 | 0.818 | 7.88 x 10^-5^ |
| Nomogram | 0.749 | 0.694 | 0.806 | 0.5 | 0.778 | 3.45 x 10^-4^ |
| Gleason | 0.617 | 0.5 | 0.741 | 0.241 | 0.64 | 8.75 x 10^-2^ |
| Fusion | 0.727 | 0.553 | 0.889 | 0.442 | 0.721 | 1.07 x 10^-2^ |
| L+N+F | 0.957 | 0.966 | 0.947 | 0.913 | 0.979 | 1.25 x 10^-21^ |
| L+N+G | 0.891 | 0.917 | 0.865 | 0.781 | 0.914 | 4.42 x 10^-10^ |
| N+F+G | 0.84 | 0.836 | 0.848 | 0.684 | 0.862 | 1.21 x 10^-5^ |
| L+F+G | 0.938 | 0.992 | 0.887 | 0.88 | 0.968 | 8.88 x 10^-18^ |
| L+N+F+G | 0.977 | 0.983 | 0.971 | 0.954 | 0.991 | 7.24 x 10^-26^ |
|  |  |  |  |  |  |  |
| **Equal split testing data (n=35)** | | | | | | |
| LSR | 0.669 | 0.594 | 0.739 | 0.333 | 0.705 | 2.49 x 10^-2^ |
| Nomogram | 0.686 | 0.676 | 0.694 | 0.371 | 0.788 | 1.12 x 10^-4^ |
| Gleason | 0.64 | 0.529 | 0.744 | 0.274 | 0.659 | 5.11 x 10^-2^ |
| Fusion | 0.768 | 0.594 | 0.9 | 0.493 | 0.747 | 4.19 x 10^-3^ |
| L+N+F | 0.829 | 0.796 | 0.855 | 0.651 | 0.921 | 2.80 x 10^-11^ |
| L+N+G | 0.743 | 0.741 | 0.744 | 0.486 | 0.8 | 2.98 x 10^-4^ |
| N+F+G | 0.755 | 0.778 | 0.733 | 0.51 | 0.847 | 2.06 x 10^-5^ |
| L+F+G | 0.773 | 0.758 | 0.787 | 0.545 | 0.903 | 7.01 x 10^-9^ |
| L+N+F+G | 0.829 | 0.755 | 0.884 | 0.639 | 0.908 | 4.91 x 10^-8^ |

L-LSR; N-Nomogram; F-fusion transcript status; G-Gleason grade;

L+N+F: LDA model to combine LSR, Nomogram and fusion transcript status;

L+N+G: LDA model to combine LSR, Nomogram and Gleason grade;

N+F+G: LDA model to combine Nomogram, fusion transcript status and Gleason grade;

L+N+F+G: LDA model to combine LSR, Nomogram, fusion transcript status and Gleason grade.
