# Supplementary material for: Genomic Copy Number Variations in the Genomes of Leukocytes Predict Prostate Cancer Clinical Outcomes
Source: PLoS One. 2015 Aug 21;10(8):e0135982. doi: 10.1371/journal.pone.0135982 (PMC4546524; doi:10.1371/journal.pone.0135982)
Supplement: S11 Table — (DOCX) [file pone.0135982.s014.docx]

| **Supplemental Table 11: Prediction of lethal prostate cancer recurrent (PSADT<4 months and relapse time <12 months) VS non-recurrence based on leukocyte LSR, Gleason, Nomogram and fusion transcript status (the representative result for Figure S2).** | | | | | | |
| --- | --- | --- | --- | --- | --- | --- |
|  |  |  |  |  |  |  |
| Model | Accuracy | Sensitivity | Specificity | Youden index | AUC | ROC p-value |
| **Equal split training data (n=35)** | | | | | | |
| LSR | 0.743 | 0.5 | 1 | 0.5 | 0.827 | 3.71 x 10^-5^ |
| Nomogram | 0.743 | 0.667 | 0.824 | 0.49 | 0.791 | 1.07 x 10^-4^ |
| Gleason | 0.571 | 0.444 | 0.706 | 0.15 | 0.592 | 3.34 x 10^-1^ |
| Fusion | 0.714 | 0.538 | 0.867 | 0.405 | 0.703 | 2.46 x 10^-2^ |
| L+N+F | 0.929 | 1 | 0.867 | 0.867 | 0.959 | 2.71 x 10^-13^ |
| L+N+G | 0.914 | 1 | 0.824 | 0.824 | 0.951 | 3.46 x 10^-14^ |
| N+F+G | 0.821 | 0.769 | 0.867 | 0.636 | 0.833 | 6.62 x 10^-4^ |
| L+F+G | 0.893 | 1 | 0.8 | 0.8 | 0.938 | 4.14 x 10^-10^ |
| L+N+F+G | 0.964 | 1 | 0.933 | 0.933 | 0.995 | < 10^-30^ |
|  |  |  |  |  |  |  |
| **Equal split testing data (n=35)** | | | | | | |
| LSR | 0.686 | 0.471 | 0.889 | 0.359 | 0.717 | 2.60 x 10^-2^ |
| Nomogram | 0.743 | 0.824 | 0.667 | 0.49 | 0.778 | 2.29 x 10^-4^ |
| Gleason | 0.686 | 0.588 | 0.778 | 0.366 | 0.722 | 7.25 x 10^-3^ |
| Fusion | 0.783 | 0.6 | 0.923 | 0.523 | 0.762 | 8.35 x 10^-3^ |
| L+N+F | 0.913 | 1 | 0.846 | 0.846 | 1 | < 10^-30^ |
| L+N+G | 0.8 | 0.765 | 0.833 | 0.598 | 0.81 | 3.14 x 10^-4^ |
| N+F+G | 0.783 | 0.8 | 0.769 | 0.569 | 0.873 | 1.30 x 10^-4^ |
| L+F+G | 0.826 | 0.9 | 0.769 | 0.669 | 0.95 | 1.06 x 10^-10^ |
| L+N+F+G | 0.87 | 0.8 | 0.923 | 0.723 | 0.892 | 8.63 x 10^-6^ |

L-LSR; N-Nomogram; F-fusion transcript status; G-Gleason grade;

L+N+F: LDA model to combine LSR, Nomogram and fusion transcript status;

L+N+G: LDA model to combine LSR, Nomogram and Gleason grade;

N+F+G: LDA model to combine Nomogram, fusion transcript status and Gleason grade;

L+N+F+G: LDA model to combine LSR, Nomogram, fusion transcript status and Gleason grade.
