# Supplementary material for: Genomic Copy Number Variations in the Genomes of Leukocytes Predict Prostate Cancer Clinical Outcomes
Source: PLoS One. 2015 Aug 21;10(8):e0135982. doi: 10.1371/journal.pone.0135982 (PMC4546524; doi:10.1371/journal.pone.0135982)
Supplement: S12 Table — (DOCX) [file pone.0135982.s015.docx]

**Supplemental Table 12: Pairwise ROC p-value for prostate cancer lethal-recurrent and non-recurrent status prediction (the geometric mean of the 10 cross-validations)**

**Training => Training**

|  | LSR | Nomogram | Gleason | Fusion | L+F+N+G | F+N+G | L+F+G | L+F+N | L+N+G |
| --- | --- | --- | --- | --- | --- | --- | --- | --- | --- |
| LSR | 1 | 6.05E-1 | 9.74E-2 | 2.21E-1 | 2.60E-2 | 3.37E-1 | 6.06E-2 | 3.94E-2 | 1.66E-1 |
| Nomogram |  | 1 | 1.19E-2 | 3.46E-1 | 8.71E-3 | 3.29E-1 | 2.47E-2 | 1.47E-2 | 4.29E-2 |
| Gleason |  |  | 1 | 2.24E-1 | 3.11E-4 | 3.25E-2 | 9.01E-4 | 5.15E-4 | 1.34E-3 |
| Fusion |  |  |  | 1 | 4.98E-4 | 6.97E-2 | 7.19E-4 | 5.85E-4 | 3.79E-2 |
| L+F+N+G |  |  |  |  | 1 | 9.06E-2 | 4.09E-1 | 5.55E-1 | 1.64E-1 |
| F+N+G |  |  |  |  |  | 1 | 1.93E-1 | 1.22E-1 | 3.72E-1 |
| L+F+G |  |  |  |  |  |  | 1 | 4.80E-1 | 3.11E-1 |
| L+F+N |  |  |  |  |  |  |  | 1 | 2.40E-1 |
| L+N+G |  |  |  |  |  |  |  |  | 1 |

L-LSR; N-Nomogram; F-fusion transcript status; G-Gleason grade;

L+N+F: LDA model to combine LSR, Nomogram and fusion transcript status;

L+N+G: LDA model to combine LSR, Nomogram and Gleason grade;

N+F+G: LDA model to combine Nomogram, fusion transcript status and Gleason grade;

L+N+F+G: LDA model to combine LSR, Nomogram, fusion transcript status and Gleason grade.

**Training => Testing**

|  | LSR | Nomogram | Gleason | Fusion | L+F+N+G | F+N+G | L+F+G | L+F+N | L+N+G |
| --- | --- | --- | --- | --- | --- | --- | --- | --- | --- |
| LSR | 1 | 3.75E-1 | 5.97E-1 | 2.47E-1 | 6.64E-2 | 1.26E-1 | 5.83E-2 | 4.39E-2 | 1.37E-1 |
| Nomogram |  | 1 | 1.30E-2 | 3.36E-1 | 1.69E-1 | 3.13E-1 | 1.29E-1 | 1.03E-1 | 4.31E-1 |
| Gleason |  |  | 1 | 1.74E-1 | 1.52E-2 | 4.21E-2 | 1.28E-2 | 8.93E-3 | 5.76E-2 |
| Fusion |  |  |  | 1 | 4.07E-2 | 2.19E-1 | 2.35E-2 | 1.20E-2 | 2.81E-1 |
| L+F+N+G |  |  |  |  | 1 | 2.31E-1 | 4.59E-1 | 4.72E-1 | 2.64E-1 |
| F+N+G |  |  |  |  |  | 1 | 3.14E-1 | 1.78E-1 | 3.49E-1 |
| L+F+G |  |  |  |  |  |  | 1 | 2.66E-1 | 2.33E-1 |
| L+F+N |  |  |  |  |  |  |  | 1 | 1.77E-1 |
| L+N+G |  |  |  |  |  |  |  |  | 1 |

L-LSR; N-Nomogram; F-fusion transcript status; G-Gleason grade;

L+N+F: LDA model to combine LSR, Nomogram and fusion transcript status;

L+N+G: LDA model to combine LSR, Nomogram and Gleason grade;

N+F+G: LDA model to combine Nomogram, fusion transcript status and Gleason grade;

L+N+F+G: LDA model to combine LSR, Nomogram, fusion transcript status and Gleason grade.
