# Supplementary material for: Genomic Copy Number Variations in the Genomes of Leukocytes Predict Prostate Cancer Clinical Outcomes
Source: PLoS One. 2015 Aug 21;10(8):e0135982. doi: 10.1371/journal.pone.0135982 (PMC4546524; doi:10.1371/journal.pone.0135982)
Supplement: S13 Table — (DOCX) [file pone.0135982.s016.docx]

| **Supplemental Table 13: Survival p-values for the predicted prostate cancer lethal-recurrent and non-recurrent groups (the geometric mean of the 10 cross-validations).** | |
| --- | --- |
|  |  |
| **Model** | **Survival p-value between two groups** |
| LSR | 5.79 x 10^-4^ |
| Nomogram | 2.79 x 10^-3^ |
| Gleason | 5.40 x 10^-2^ |
| Fusion | 9.26 x 10^-4^ |
| LSR + Nomogram + Fusion | 1.24 x 10^-5^ |
| LSR + Nomogram + Gleason | 5.12 x 10^-5^ |
| Nomogram + Fusion + Gleason | 3.49 x 10^-4^ |
| LSR + Fusion + Gleason | 2.37 x 10^-4^ |
| LSR + Nomogram + Fusion + Gleason | 4.24 x 10^-6^ |
