# Supplementary material for: Genomic Copy Number Variations in the Genomes of Leukocytes Predict Prostate Cancer Clinical Outcomes
Source: PLoS One. 2015 Aug 21;10(8):e0135982. doi: 10.1371/journal.pone.0135982 (PMC4546524; doi:10.1371/journal.pone.0135982)
Supplement: S14 Table — (DOCX) [file pone.0135982.s017.docx]

**Supplemental Table 14: Pairwise survival p-value for prostate cancer lethal-recurrent and non-recurrent status prediction (the geometric mean of the 10 cross-validations)**

|  | LSR | Nomogram | Gleason | Fusion | L+F+N+G | F+N+G | L+F+G | L+F+N | L+N+G |
| --- | --- | --- | --- | --- | --- | --- | --- | --- | --- |
| LSR | 1 | 4.41E-2 | 6.20E-3 | 6.16E-2 | 1.48E-3 | 4.87E-2 | 3.56E-2 | 3.67E-3 | 2.89E-2 |
| Nomogram |  | 1 | 3.22E-2 | 1.17E-1 | 7.15E-4 | 4.34E-2 | 1.90E-2 | 2.12E-3 | 8.86E-3 |
| Gleason |  |  | 1 | 9.72E-3 | 4.50E-5 | 3.82E-3 | 2.50E-3 | 1.25E-4 | 5.26E-4 |
| Fusion |  |  |  | 1 | 1.56E-3 | 4.12E-2 | 3.79E-2 | 4.76E-3 | 2.75E-2 |
| L+F+N+G |  |  |  |  | 1 | 3.15E-3 | 8.83E-3 | 6.16E-2 | 1.07E-2 |
| F+N+G |  |  |  |  |  | 1 | 2.46E-2 | 6.80E-3 | 5.16E-2 |
| L+F+G |  |  |  |  |  |  | 1 | 2.54E-2 | 6.61E-2 |
| L+F+N |  |  |  |  |  |  |  | 1 | 2.80E-2 |
| L+N+G |  |  |  |  |  |  |  |  | 1 |

L-LSR; N-Nomogram; F-fusion transcript status; G-Gleason grade;

L+N+F: LDA model to combine LSR, Nomogram and fusion transcript status;

L+N+G: LDA model to combine LSR, Nomogram and Gleason grade;

N+F+G: LDA model to combine Nomogram, fusion transcript status and Gleason grade;

L+N+F+G: LDA model to combine LSR, Nomogram, fusion transcript status and Gleason grade.
